# Exposure history determines pteropod vulnerability to ocean acidification along the US West Coast

**DOI:** 10.1038/s41598-017-03934-z

**Published:** 2017-07-03

**Authors:** N. Bednaršek, R. A. Feely, N. Tolimieri, A. J. Hermann, S. A. Siedlecki, G. G. Waldbusser, P. McElhany, S. R. Alin, T. Klinger, B. Moore-Maley, H. O. Pörtner

**Affiliations:** 1 Pacific Marine Environmental Laboratory, National Oceanic and Atmospheric Administration, 7600 Sand Point Way NE, Seattle, WA 98115 USA; 2 Southern California Coastal Water Research Project (SCCWRP), 3535 Harbor Blvd # 110, Costa Mesa, CA 92626 USA; 3 Conservation Biology Division, Northwest Fisheries Science Center, National Marine Fisheries Service, National Oceanic and Atmospheric Administration, 2725 Montlake Boulevard E, Seattle, Washington 98112 USA; 4 University of Washington, Joint Institute for the Study of the Atmosphere and Ocean (JISAO), 3737 Brooklyn Avenue NE, Box 355672, Seattle, WA 98195 USA; 5 Oregon State University, College of Earth, Ocean, and Atmospheric Sciences, 101 SW 26th Street, Corvallis, OR 97331 USA; 6 School of Marine and Environmental Affairs, University of Washington, 3707 Brooklyn Avenue NE, Seattle, WA 98105 USA; 7 University of British Columbia, Department of Earth, Ocean, and Atmospheric Sciences, Vancouver, BC V6T 1Z4 Canada; 8 Alfred Wegener Institute, Postfach 12 01 61, Am Handelshafen 12, 27570 Bremerhaven, Germany

## Abstract

The pteropod *Limacina helicina* frequently experiences seasonal exposure to corrosive conditions (Ω_ar _ < 1) along the US West Coast and is recognized as one of the species most susceptible to ocean acidification (OA). Yet, little is known about their capacity to acclimatize to such conditions. We collected pteropods in the California Current Ecosystem (CCE) that differed in the severity of exposure to Ω_ar_ conditions in the natural environment. Combining field observations, high-CO_2_ perturbation experiment results, and retrospective ocean transport simulations, we investigated biological responses based on histories of magnitude and duration of exposure to Ω_ar_ < 1. Our results suggest that both exposure magnitude and duration affect pteropod responses in the natural environment. However, observed declines in calcification performance and survival probability under high CO_2_ experimental conditions do not show acclimatization capacity or physiological tolerance related to history of exposure to corrosive conditions. Pteropods from the coastal CCE appear to be at or near the limit of their physiological capacity, and consequently, are already at extinction risk under projected acceleration of OA over the next 30 years. Our results demonstrate that Ω_ar_ exposure history largely determines pteropod response to experimental conditions and is essential to the interpretation of biological observations and experimental results.

## Introduction

Over the last two-and-a-half centuries, the global oceans have absorbed about ~550 billion tons of anthropogenic carbon dioxide (CO_2_) emissions released into the atmosphere from the burning of wood or fossil fuels for energy, land-use changes, and from cement production^1, 2^. This absorption of atmospheric CO_2_ has increased ocean acidity in a process referred to as ocean acidification (OA). The California Current Ecosystem (CCE) is particularly vulnerable to OA because it naturally experiences corrosive conditions (i.e., undersaturated waters with respect to aragonite, Ω_ar_ < 1) due to seasonal upwelling. Since the beginning of the industrial era, steadily increasing atmospheric CO_2_ levels have pushed the system rapidly away from its historic range of CO_2_ conditions^3–5^, resulting in a shoaling of the Ω_ar_ saturation horizon by about 40–50 m and increasing the presence of Ω_ar_ < 1 waters in the upper water column^3, 6–9^. For many marine calcifiers, and in particular pteropods, this results in more intense and extended exposure to corrosive conditions, reducing the availability of suitable habitat and increasing their vulnerability to OA^9^.

Pteropods are a group of pelagic aragonitic calcifiers that can play an essential role in biogeochemical cycling and food web function in highly productive upwelling regions such as the CCE^10^. Pteropods are a prey species for ecologically, economically, and culturally important fish species, such as pink, chum, and sockeye salmon, as well other pelagic and demersal fish such as cod, herring, and mackerel^11–14^. There is considerable concern that one of the most dominant pteropod species, *Limacina helicina*, has already been negatively affected by OA in its natural environment through increased shell dissolution and associated changes in vertical distribution^9, 15^. These responses are closely associated with *in situ* carbonate chemistry conditions, making pteropods a sensitive bioindicator for evaluating early impacts of OA^9^. Pteropods exposure to the natural background of hypercapnic waters combined with the anthropogenic signal strengthens the exposure to OA and may thereby lead to early warning signals under moderate degrees of climate change.

Significant changes in pteropod habitat in the CCE have mainly occurred in the last few decades^16, 17^. It is reasonable to expect that exposure to progressive changes over relatively short time scales can either exacerbate vulnerability or trigger individual acclimatization, thereby enhancing individual physiological tolerance traits or diminishing sensitivity^18^. The balance between tolerance and vulnerability will ultimately determine population growth rates, persistence, and risk of extirpation or extinction. A key question is whether and how quickly organisms can compensate for negative effects of OA, either by short-term acclimatization or long-term evolutionary adaptation over generations^19^. On shorter time scales, organisms can develop the capacity to maintain performance across a range of environmental conditions through the process of acclimatization. Pteropods are exposed to a range of *in situ* Ω_ar_ conditions during their daily vertical migration and transport by currents within the CCE. Given their history of exposure to low Ω_ar_ conditions in their natural environment, we hypothesized that pteropods may be pre-adapted or have developed a physiological capacity to acclimatize to OA conditions, allowing these organisms to maintain critical biological processes regardless of the exposure history. The inability to maintain the required level of biological responses would, on the other hand, suggests a lack of adaptive capacity, and therefore the potential for vulnerability to OA.

To better understand acclimatization capacity in pteropods in the CCE, we conducted experiments to test the ability of individual pteropods to successfully maintain biological processes, including calcification and survival, following different histories of exposure to low Ω_ar_ in the natural environment. Calcification was measured across a range of *in situ* Ω_ar_ conditions from which the individuals were collected, and survival was tested under experimental conditions in which the partial pressure of CO_2_ (pCO_2_) was elevated. To best approximate pteropod exposure to Ω_ar_ conditions in the natural environment of the CCE during their diel vertical migration (DVM) in the upper 100 m^9, 15^, we chose a relatively narrow range of pCO_2_ levels from 400 to 1200 µatm (2.2 ≥ Ω_ar_ ≥ 0.8 for this set of experiments). The choice of pCO_2_ experimental conditions was aimed at delineating subtle responses and to allow comparison with prior studies performed under similar Ω_ar_ conditions that demonstrated negative physiological responses to these conditions in the laboratory (but not *in situ*)^20–23^. Also, the study was designed to determine whether and how exposure history can cause differential responses of pteropods and whether acclimatization capacity becomes apparent through shifting tolerance thresholds under variable levels of Ω_ar_ conditions during different exposure histories.

Exposure history can be examined through the most recent *in situ* conditions that the organisms experienced at the time of the collection (referred to as *in situ* Ω_ar_ conditions), or through more extended conditions (referred to as prolonged exposure) over several months, which can be simulated using a high-resolution regional model of OA conditions with a severity index. We characterized variability of exposure history using the approach described by Hauri *et al*.^5^, where the metric of exposure severity depends on both the magnitude and duration of exposure to corrosive conditions. In this study, we investigated the extent to which pteropods changed the capacities for calcification and survival as an indication of acclimatization to OA conditions. Calcification capacity, defined as the proportional glow of calcein in the shell, was inferred in relation to Ω_ar_ conditions experienced by the individual immediately prior to specimen collection. Survival capacity was determined in relation to the model-derived exposure history. This approach allowed biological responses to be considered on the timescales of natural variability of Ω_ar_ conditions in the environment that potentially influence acclimatization capacity. Moreover, understanding the role of exposure history enhances the likelihood of correctly interpreting the results of OA perturbation experiments, as well as supporting more comprehensive assessments of pteropod vulnerability, particularly with respect to the role of pteropods as effective bioindicators for OA^9^.

## Results

### Carbonate chemistry and food availability

**Figure 1:**
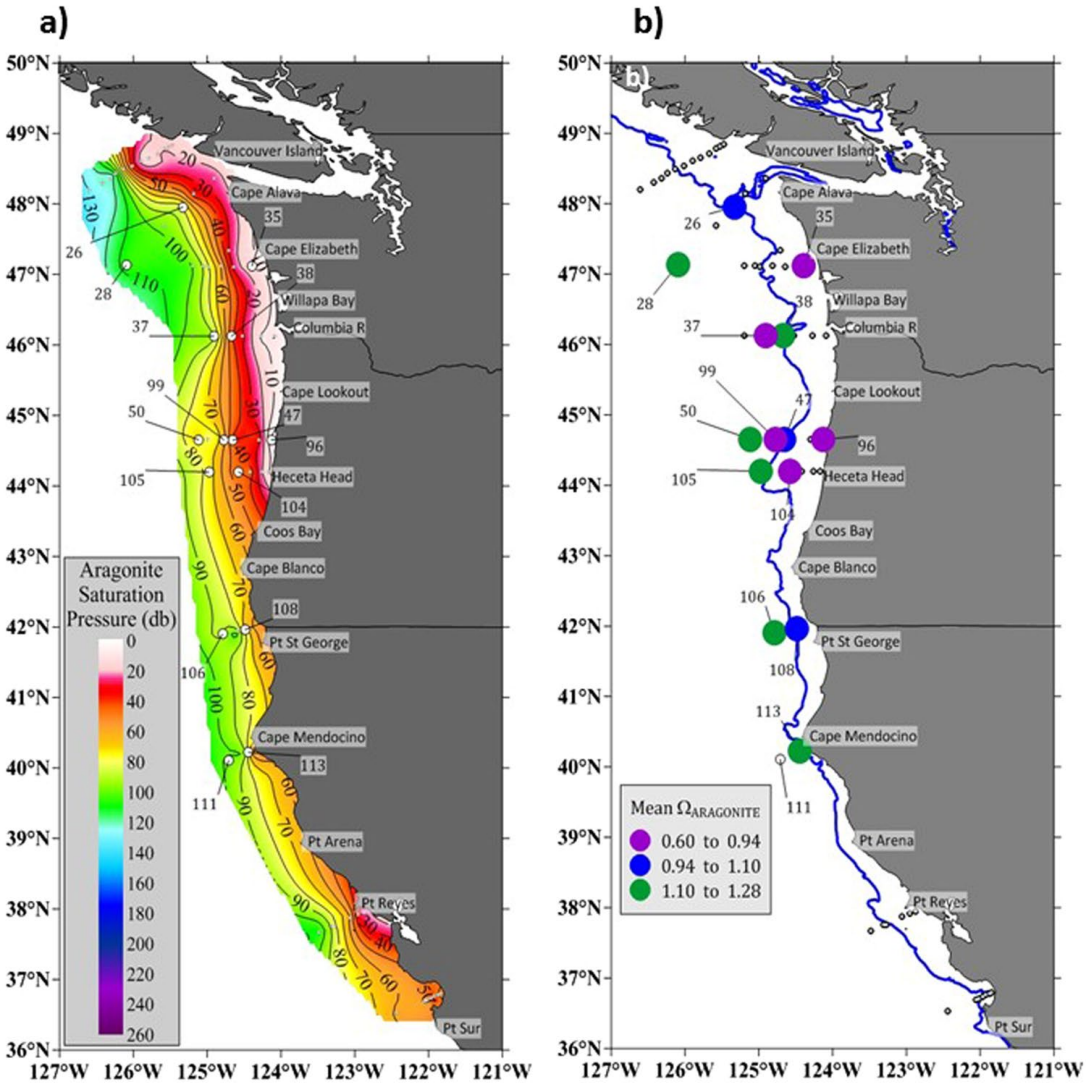
Ocean acidification (OA) conditions displayed as carbonate chemistry gradients along the US West Coast (Washington-Oregon-California). (**a**) OA is represented as the depth of aragonite saturation state (Ω_ar_), with the Ω_ar_ isocline of 1. (**b**) Along these carbonate gradients in the California Current Ecosystem, biological samples were taken at selected stations (numbers) with *in situ*  Ω_ar_ conditions corresponding to mean Ω_ar_ in the upper 100 m water column (filled circles, mean value ranges indicated in legend). Blue line indicates 200 m isobaths delineating coastal region of low Ω_ar_ conditions on the shelf and offshore with higher Ω_ar_. The figure was created using Surfer 13 Golden Software (http://www.goldensoftware.com/products/surfer).

In August 2013, shoaling of upwelled water that was undersaturated with respect to aragonite was observed to be very close to the surface ( < 20 m depth) off the coast of Washington, Oregon, and northern California (Fig. 1a). Compared to previous observations made in the spring of 2007 and the late summer of 2011^3, 9^, we observed in 2013 an intensification of undersaturated conditions in some nearshore waters (isobaths < 200 m) regions (Fig. 1b). A large fraction of the water column was exposed to corrosive conditions (Fig. 1), especially in the nearshore regions near the Columbia River and along the central Oregon coast, with strong evidence for upwelling of undersaturated water all the way to the surface in some locations. During the upwelling season (late spring through early fall) in the Pacific Northwest, the CCE is very productive and chlorophyll concentrations are high across the entire shelf (to 200 m depth) (Supplementary Fig. S7). The abundance and distribution of phytoplankton indicates that food is not a limiting parameter over the entire shelf region, whereas reduced chlorophyll concentrations are available in the offshore regions.

**Figure 2:**
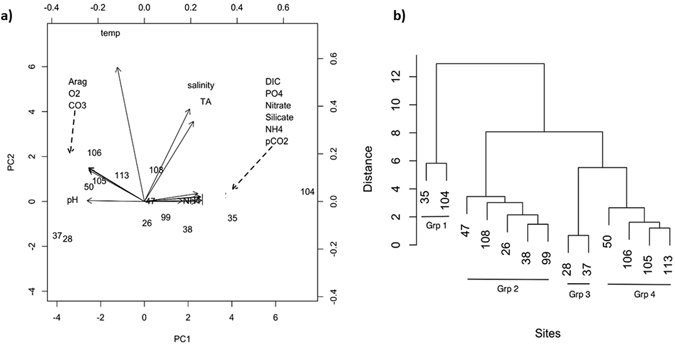
Relationships among collection sites in terms of water chemistry in the natural environment using principal coordinate’s analysis and cluster analyses. (**a**) The environmental variables were highly correlated, with multicollinearity between different parameters (e.g., between carbonate ion, Ω, and O_2_) and thus, loading overlap on the left figure (dashed arrows indicating overlapping symbols). (**b**) Cluster analyses of the water chemistry at the stations of pteropod collection showed similarity among the stations in environmental parameters, and resulted in four clearly identified groups: Group 1 (stations 35 and 104); Group 2 (stations 47, 108, 26, 38, and 99); Group 3 (stations 28 and 37); and Group 4 (stations 50, 106, 105, and 113), with average Ω_ar _~ 1.2 for Groups 3 and 4, Ω_a _~ 1 for Group 2, and Ω_ar _~ 0.75 for Group 1 (Supplementary Table S3). Biological responses were analyzed based on these cluster groupings.

Most physical and chemical parameters were highly correlated among stations (Supplementary Fig. S1 and Table S1). Principal component (PC) analysis, used to examine the relationship of environmental parameters among sites, showed that the first two PCs explained approximately 92% of the variation among sites (79% and 13%, respectively; Fig. 2 and Supplementary Table S2). Most water chemistry variables were highly correlated with the first PC axis. For example, pCO_2_ and pH both loaded on PC1 with opposite signs, indicating pH decrease with increasing pCO_2_. Cluster analysis to evaluate similarities among the stations in background environmental parameters revealed four main groupings that differed in mean Ω_ar_ (Fig. 2b). Based on the differences in Ω_ar_ between clusters, differential pteropod responses were assigned to individual clusters characterized by the Ω_ar_ thresholds of each.

### Particle tracking

**Figure 3:**
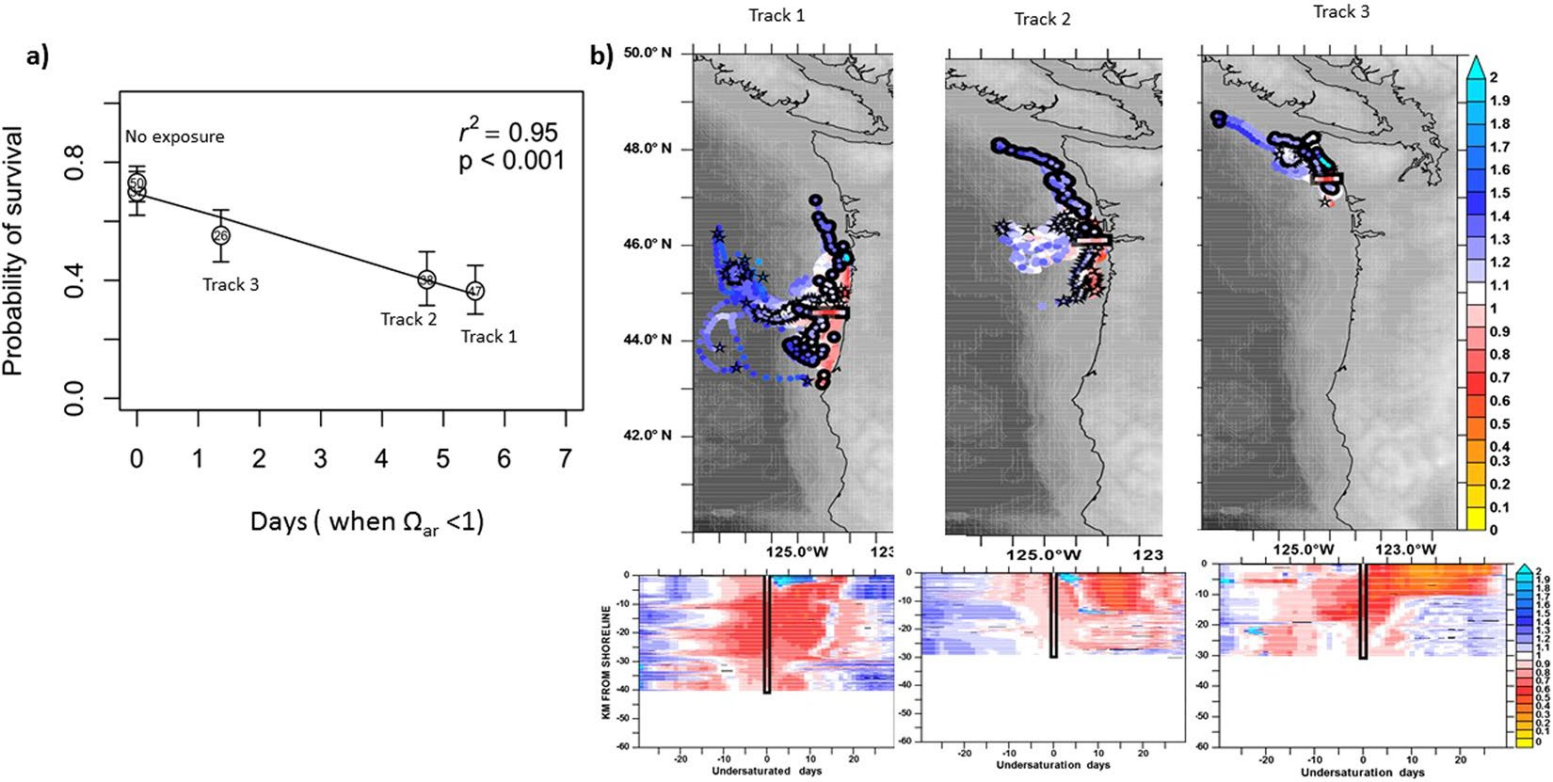
Exposure history to low Ω_ar_ in the natural environment and its impact on the probability of survival. (**a**) Survival probability was determined by taking into account pteropod exposure history over the last 5–6 weeks in the CCE, based on the model outputs (**b**). The survival probability was highest at the offshore stations with no exposure history (stations 37, 50, and 106), followed by a decline at stations of “moderate” exposure (undersaturation days of ~1.5 at stations 26 and 28; exposure conditions represented on Track 3), and the lowest survival probability at the most severe exposures (undersaturation days of ~5 for stations 47 and 38; exposure conditions represented on Tracks 1 and 2, respectively). Station number is shown inside each circle. Tracks not shown for the offshore station. (**b**) Daily averaged spatial tracks and time history of aragonite saturation state for modeled particles released 1 August 2013 along cross-shelf lines spanning 126.0°W to 123.5°W, and tracked both forward and backward in time to span 1 July–31 August 2013. Particle tracking includes vertical migration of particles between 10 and 100 m. For each track we show the instantaneous values of aragonite saturation state (indicated by shading); these are further illustrated by the Hovmöller diagrams of saturation history for all tracked particles as a function of days since 1 August 2013 (x-axis) and their cross-shelf location on the release date (y-axis). Track 1 (station 47) covers a cross-shelf line near Heceta Head (44.6°N) and Track 2 (station 38) covers a cross-shelf line off Columbia River (46.2°N); both tracks are characterized by severe exposure (0.6 ≤ Ω_ar_ ≤ 0.8) for approximately 3–4 weeks). Track 3 (stations 26 and 28) was released just off 47.7°N with exposure to less severe conditions (0.8 ≤ Ω_ar_ ≤ 1) for approximately 2 weeks. Circles indicate start locations of a representative subsample of the tracks on 1 July 2013; rectangles indicate their location on 1 August 2013; stars indicate their end locations on 30 August 2013. The figure was created using Ferret version 6 (http://www.ferret.noaa.gov/Ferret/).

Model-derived severity expressed as undersaturation days (see *Methods*, equations (1) and (2)) provides an integrated exposure metric representative of pteropod exposure history in their natural environment. Spatial maps show the paths and saturation histories of DVM particles released along transects coincident with observed sampling efforts (Fig. 3b). The particle trajectories themselves reflect conditions typical of upwelling for the Pacific Northwest, with predominantly southward alongshore flow during summer. Particles released off Oregon (Newport Line) and off the Columbia River (Fig. 3b, Tracks 1 and 2) within 60 km of the coast were exposed to more severe undersaturated conditions (0.6 ≤ Ω_ar_ ≤ 0.8) for approximately 4–5 weeks prior to our field sampling. These stations (47 and 38) also experienced the highest cumulative undersaturation days (the value of ~5; Fig. 3a). Off Washington, particles passing the nearshore shelf (within 10–20 km) experienced moderate undersaturated conditions (0.8 ≤ Ω_ar_ ≤ 1) for approximately 2–3 weeks prior to sampling (Fig. 3b, Track 3); their severity index of cumulative undersaturation days is lower with values ~2 (stations 26 and 28; Fig. 3a). The particles released more than 80 km from the coast did not encounter Ω_ar_ ≤ 1 during the simulation period (the value of undersaturated days is zero; Fig. 3a). These findings suggest that pteropods transported along with the horizontal currents with DVM between 10 and 100 m were likely collected in local water chemistry that was representative of the pteropods’ recent exposure for approximately 5–6 weeks.

### Calcification linked to *in situ* carbonate chemistry

**Figure 4:**
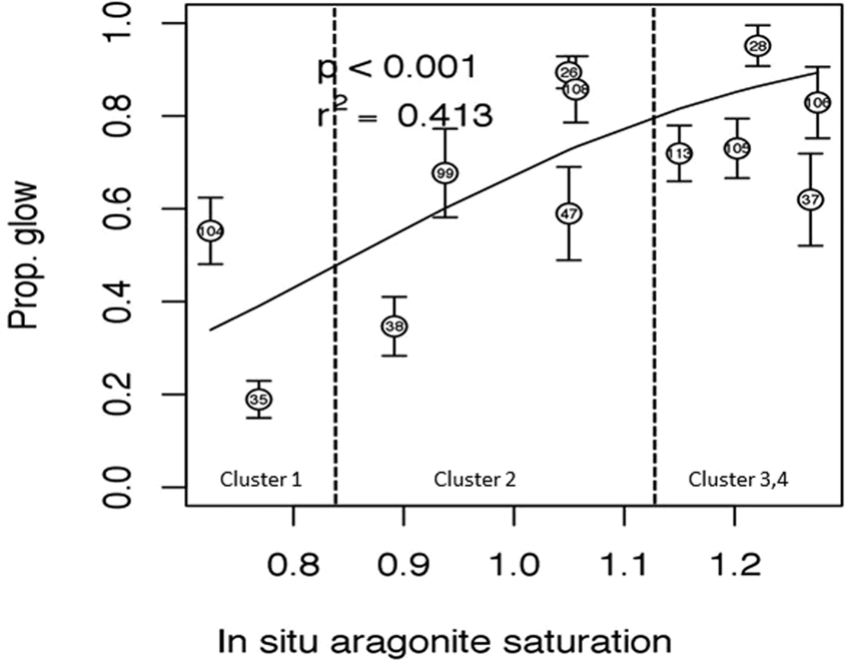
The changes in pteropod calcification depicted as a proportional glow, at the range of *in situ* conditions from 12 different stations characterized by the difference in carbonate chemistry. The regression line from a general linear model with logit-link and beta error distribution indicates positive correlation between aragonite saturation (Ω_ar_) and proportional glow. Pteropods coming from high *in situ* Ω_ar_ demonstrate high glow of calcein in their shell, shown also visually in Fig. 5a and 5b Vertical lines indicate groupings based on the cluster analysis of stations; Cluster Group 1 (Ω_ar_ < 0.8; stations 35 and 104), Cluster Group 2 (0.8 < Ω_ar_ < 1; stations: 47, 108, 26, 38, 99), and Cluster Group 3 and 4 (Ω_ar_ > 1.2; stations: 28, 37, 106, 105, 113). Error bars indicate ± 1.0 standard errors. The average *in situ* Ω_ar_ of three groupings identifies two aragonite saturation state thresholds with respect to proportional glow; Ω_ar_ > 1.2 and Ω_ar_ < 0.8. Error bars indicate ± 1.0 standard errors, N = 96 (Table S3). Proportional glow and Ω_ar_ are related through the following equation: ln (% glow/(1-% glow)) = b_0_ + b_1_*(Ω_ar_) = −4.34( ± 0.19 s.e.) + 5.07( ± 0.18 s.e.)* Ω_ar_.

**Figure 5:**
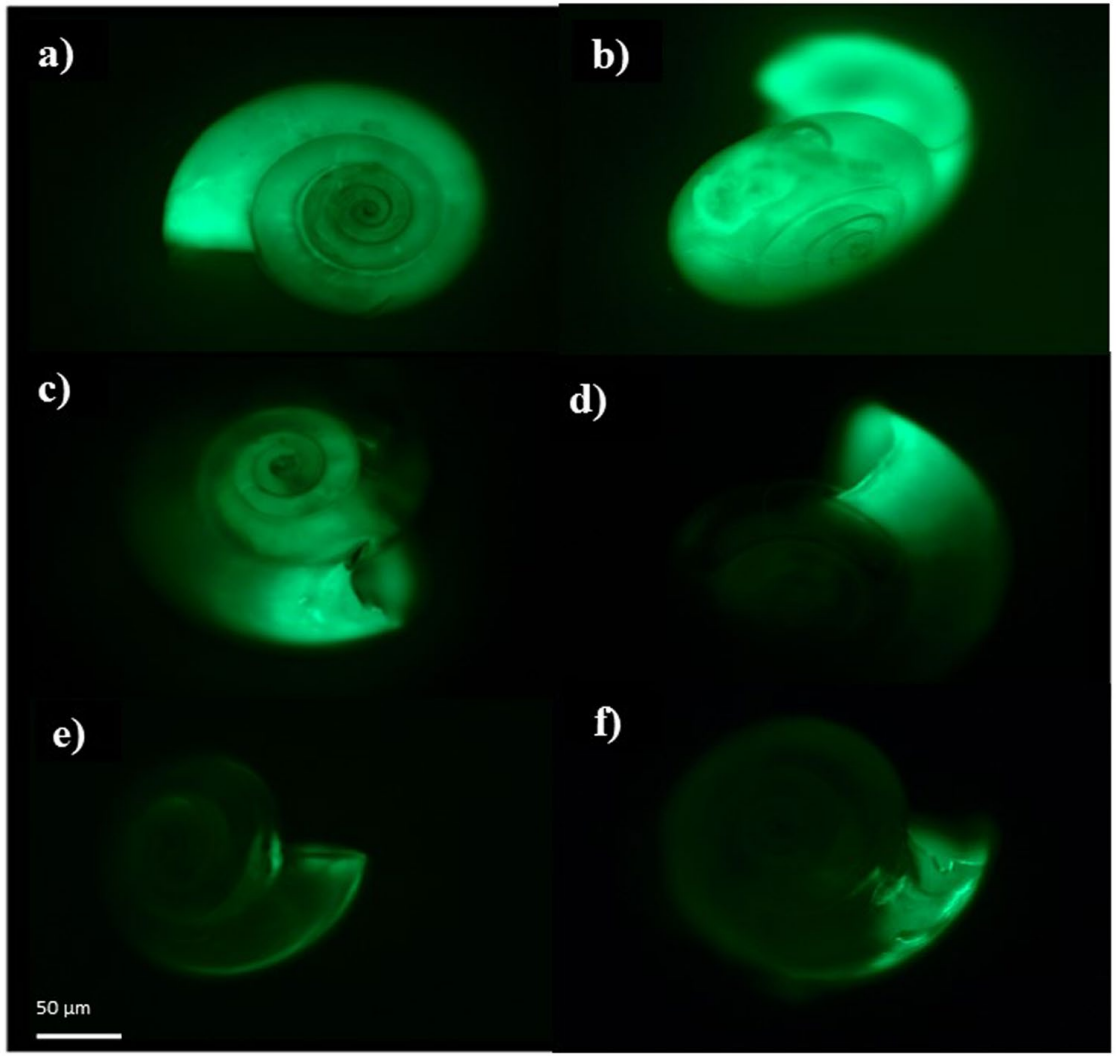
Calcification patterns in *Limacina helicina* depicted as the proportional fluorescent glow of incorporated calcein dye under the epifluorescence microscope. The greatest extent of fluorescent glow indicates the most active calcification activity and was present in pteropods collected from Cluster Groups 3 and 4 (with Ω_ar _ ≥ 1.2; **a** and **b**) and occurs over the entire shell, from the protoconch to the growing edge. At lower Ω_ar_ (Cluster Group 2; 0.8 < Ω_ar_ < 1.2) calcification extent is reduced to more distal parts of the shell (**c** and **d**), while overall decline in calcification occurs in pteropods from the Cluster Group 1 (Ω_ar_ < 0.8; **e** and **f**) with only parts of the growing edge still showing evidence of calcification activity. Pteropods were exposed to calcein dye for 18–20 hours; the images were taken at the same magnification (10 × ) and the scale bar (50 µm) indicates the size of the animal.

Calcification capacity (quantified as the proportion of fluorescing shell surface) was linked with coincident observations of spatially variable ocean carbonate conditions and food availability in order to highlight spatial variability of the calcification response in this region. Calcification proportion differed among pteropods collected at different stations (step one, best-fit model included stations: Akaike Information Criteria (AICc_null_) = −8.86, AICc_stations_ = −65.78, and this variation was related to *in situ* carbonate chemistry conditions with the best-fit model including Ω_ar_ and total alkalinity (TA) and pH residuals (Supplementary Table S4), where Ω_ar_ also explained 41% of the variation in calcification among stations (Fig. 4 and Supplementary Tables S4 and S5; r^2^ = 0.413, p = 0.001). In general, calcification was highest at locations where the degree of supersaturation was highest (Cluster Group 3, 4; Ω_ar _~ 1.2; Fig. 5a,b) and the lowest in the undersaturated conditions (Ω_ar_ ~0.75; Cluster Group 1; Fig. 5e,f). Moreover, model comparison via AIC showed that the aragonite-only model was always the best-fit model, whether or not chlorophyll concentrations (i.e., food availability) were included. In offshore regions, calcification depended only on Ω_ar_ and was not affected by low chl-a concentrations. In the nearshore regions, however, the observations with lower AIC value for the chl-a-only model compared to the null model suggest that food availability might be important for calcification, although not of primary importance (Supplementary Table S12). The best correlation between calcification and chl-a concentrations was obtained by tracking the exposure history to food availability over the previous 5–6 weeks (Supplementary Tables S12 and S13).

High calcification (% glow) at Ω_ar_ > 1 (Cluster Groups 3 and 4) was revealed by the intense glow over the entire shell, indicative of the ongoing process of thickening throughout the shell and extending the shell at the growing edge (Fig. 5a,b). At near-saturated conditions (Ω_ar_ ~1), we observed the proportional glow to be reduced, indicating the location of calcification shifting from the entire shell surface to the growing edge, and ultimately leading to the loss of shell thickness (Cluster Group 2; Fig. 5c,d, and Supplementary Figs S4 and S5). In addition, the mean intensity of the fluorescence glow at the growing edges was reduced, indicating that shell growth was also declining. Rather than maintaining calcification under physicochemical conditions unfavorable for building shells, pteropods respond through a decline in calcification and growth. This demonstrates that pteropods do not have the capacity to maintain calcification across carbonate chemistry gradients at low Ω_ar_ values, unless surface water chlorophyll concentrations at a selected station are present in very high concentrations (Station 104 with surface chl-a of 28.74 mg/L; Supplementary Table S13). Although the approximate threshold of water mean aragonite saturation state around Ω_ar_ ≥ 1.2 still allows for continuation of the calcification process (Fig. 5a,b), this can be considered the primary apparent threshold for declining calcification and growth (called apparent as the physicochemistry in the calcification compartment is the one directly driving calcification). Percent glow was greatly reduced or absent at Ω_ar_ < 0.8 (Cluster Group 1; Figs 4 and 5e,f, and Supplementary Table S4), indicating a secondary threshold for calcification.

### Linking survival to exposure history

Survival success was an end-point measure in the experiments where pteropods with different exposure histories were tested for their capacity to maintain survival at experimental high-CO_2_ conditions. Through the comparison of biological responses among stations and across pCO_2_ experimental treatments, we were able to examine similarities between survival patterns that occurred under pCO_2_ concentrations in the natural environment and under comparable experimental conditions. Using a relatively narrow range of pCO_2_ treatments allowed us to establish with more confidence the levels at which effects on survival become apparent.

**Figure 6:**
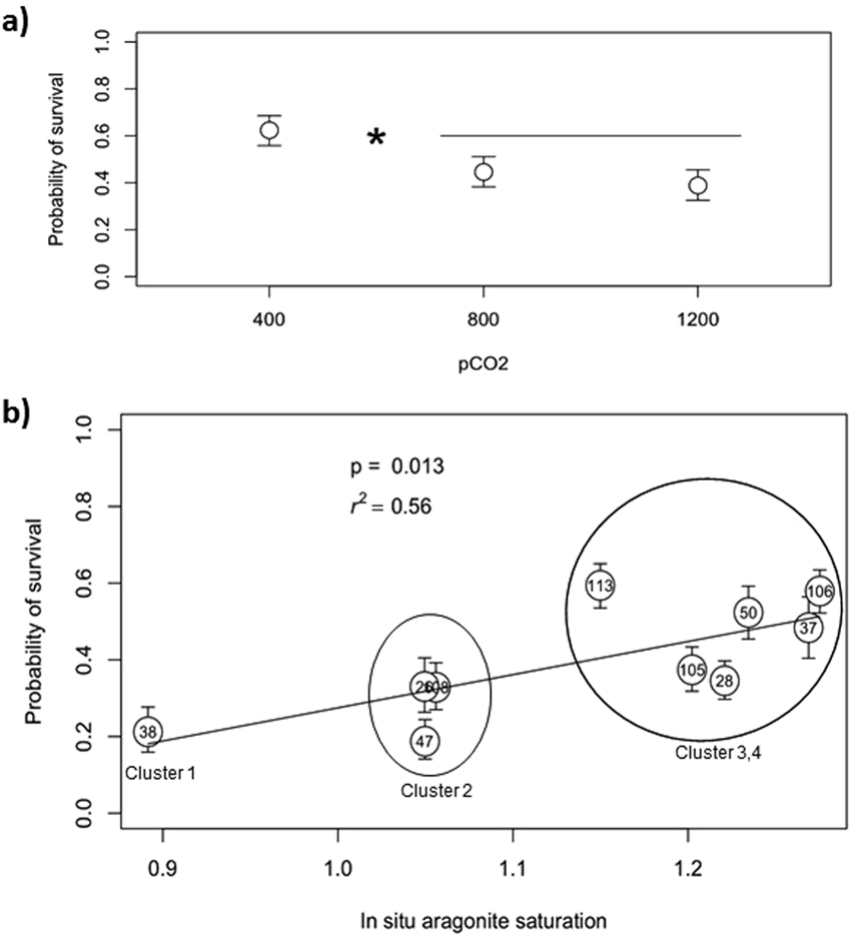
Probability of survival in *Limacina helicina* in the high CO_2_ experimental setup with consideration given to underlying *in situ* Ω_ar_ exposure at the collection site. (**a**) Probability of survival based on experimental pCO_2_ is the highest in the lowest pCO_2_ treatment regardless of the exposure history. N_400_ = 187; N_800_ =186; N_1200_ =197. (**b**) Relationship between Ω_ar_ at the station of collection and survival with pteropods maintained at 1200 µatm experimental conditions. The highest survival probability was found at the sites with the most favorable conditions (shown here in Cluster Groups 3 and 4 (Ω_ar_ ≥ 1.2)). Survival declined under less favorable conditions (shown here in Cluster Group 2 (0.8 ≥ Ω_ar_ ≥ 1.2) and Cluster Group 1 (Ω_ar_ ~ 0.89)). This linear relationship between survival probability and Ω_ar_ is described as function = b_0_ + b_1_*(Ω_ar_) = −0.47 ( ± 0.31 s.e.) + 0.92( ± 0.27 s.e.)* Ω_ar_. Error bars indicate ± 1.0 standard errors. N = 570 (Supplementary Table S7). Numbers within circles identify the station.

Pteropod survival was related to the station of collection and declined with increasing experimental pCO_2_, but there was no simultaneous interaction between the two factors (Fig. 6a and Supplementary Table S9). Survival at baseline conditions (400 μatm) was 1.6 times (or ~40%) higher than at 1200 μatm but no difference was observed between the 800 and 1200 pCO_2_ treatments (Fig. 6a and Supplementary Table S10). The Ω_ar_ threshold for survival, beyond which significantly lower values were seen, was identified at 800 µatm treatment (Ω_ar_ ~1.05 ± 0.2). This observation demonstrated that even small changes from supersaturated to near-saturation Ω_ar_ can affect pteropod survival probability. The lack of an interaction between experimental pCO_2_ and station of collection indicates that the pattern of survival among stations was the same as across the pCO_2_ treatments, an important consideration when assessing acclimatization. Therefore, we used means for the stations from the 400 μatm treatment to determine whether *in situ* environmental conditions explained variation in survival among stations. The model including Ω_ar_ explained 60% of the probability of survival for pteropods among stations (Fig. 6b and Supplementary Table S10), with survival decreasing at lower *in situ* Ω_ar_ conditions. Inclusion of other parameters (of carbonate chemistry and temperature) did not improve the model fit (Supplementary Table S11). The identified threshold for high survival was identified to be at a value of Ω_ar _~ 1.2 (average Ω_ar_ in Cluster Groups 3 and 4), followed by a 47% decrease in survival probability with declining Ω_ar_ (Cluster Groups 1 and 2; Fig. 6b) between cluster groups. Food availability does not have an impact on survival probability. As evidenced by the lowest AIC value, model comparison shows that Ω_ar_ alone is the best predictor of survival (Supplementary Table S12). High concentrations of chl-a present in the nearshore and shelf regions did not increase survival probability, and thus did not offset the impact of OA conditions or provide the organisms with additional acclimatization potential. In addition to *in situ* Ω_ar_, the impact of exposure duration to Ω_ar_ < 1 over recent (5–6 weeks) history on probability of survival in the high CO_2_ experiments was investigated. Model particle output (severity index quantified as undersaturation days, see *Methods*) overlaid with *in situ* conditions at pteropod origin showed a significant negative correlation (r^2^ = 0.87; Supplementary Fig. S6). The number of undersaturation days explained almost 90% of survival within treatment (Fig. 3a), which was notably better when just the *in situ* conditions without the duration were considered (Fig. 6b; R^2^ = 0.6). We found a detectable decline in survival probability in experimental conditions in the individuals with prolonged exposure to Ω_ar_ < 1 conditions; this pattern was most pronounced at the stations with the most severe exposure to low Ω_ar_ (0.6 ≤ Ω_ar_ ≤ 0.8) lasting approximately 4–5 weeks (i.e., ~5 cumulative undersaturation-days for the samples at stations 38 and 47; Fig. 3b, Tracks 1 and 2), followed by 2–3 week exposure to moderate Ω_ar_ (0.8 ≤ Ω_ar_ ≤ 1; i.e., ~2 cumulative undersaturation-days for the samples at stations 26 and 28; Fig. 3b, Track 3). The highest probability of survival was found in pteropods sampled offshore with no recent exposure to Ω_ar_ < 1 (Fig. 3a).

We conclude that survival probability is dependent on previous exposure history to low Ω_ar_, and decreases with increasing severity of exposure. Because prior exposure does not generate acclimatization, these results indicate that *L*. *helicina* lacks the capacity to increase its tolerance or diminish its sensitivity to OA.

## Discussion

This interdisciplinary study integrates experimental work, particle tracking within a realistic high-resolution numerical ocean simulation and *in situ* chemical observations. While high-CO_2_ experimental work and observations are commonly used approaches, combining field observations, experimental and modeling approaches for marine plankton is still an underutilized method for data synthesis. The modeling aspect based on *L*. *helicina*’s exposure history provided an outstanding opportunity for a more comprehensive and complex interpretation of experimental results and *in situ* observations, which would otherwise remain limited. Overall, the combination of data from all three approaches (*in situ* collection conditions, particle tracking history, and experiments) allowed us to assess the role and importance of exposure history on acclimatization potential. Our results reveal that the magnitude of exposure might be a primary causative factor in physiological response (explains an 60% of variance), while the duration of exposure is a significant factor but possibly of secondary importance (explains ﻿an additional 30%). Therefore, we strongly recommend including information on the history of exposure in future experimental studies; otherwise, the interpretation of vulnerability remains limited, to the point of possibly being erroneous.

The organisms that were able to better maintain their survival under elevated CO_2_ experimental conditions originated from the natural environment with conditions of Ω_ar_ > 1. Such conditions were most common in offshore environments; in our study, none of the pteropods from inshore stations with a prior history of exposure to low Ω_ar_ demonstrated survival capacity equivalent to that of offshore specimens. Our results suggest that pteropod responses are dependent on the extent to which an individual’s physiological status has been compromised by exposure to Ω_ar_ < 1. Our findings demonstrate that even small changes from super- to near-saturated conditions in the natural environment^24^ can affect pteropod survival. We interpret this as confirmation that the addition of CO_2_ generates physiological stress that is not reduced or relieved by acclimatization. There was a negative relationship between Ω_ar_ and the capacity to acclimatize, and no indication that history of previous exposure to low Ω_ar_ initiated any acclimatization or enhanced physiological tolerance. Overall, our findings indicate low tolerance of pteropods to OA under field conditions, and very limited capacity if any to compensate for acute exposure and acclimatize to extended periods of OA. Given that the Ω_ar_ threshold for survival was found to be around 800 µatm (Ω_ar _~ 1.05 ± 0.2), which already partially characterizes pteropod vertical habitat and will dominate over the entire coastal water column by the summer of 2050^8^, pteropods might be under continuous risk under projected acceleration of OA over the next 30 years^8^.

Reduced calcification and survival as well as developmental delays in response to low Ω_ar_ have been repeatedly reported across a variety of pteropod species originating from different locations^22, 23, 25–29^. Among studies, differing intercepts in observed responses suggest differing biological end-points, consistent with expectations of inherent natural variability among species. Moreover, many research studies are characterized by high variance (e.g., ref. 23), which can confound interpretations and make comparisons among observational studies and modeling efforts more challenging. To aid interpretation, Busch *et al*.^23^ recommend that investigators include experience prior to capture before starting experiments.

With respect to diminished calcification capacity coinciding with low Ω_ar_ exposure, we observed patterns that differed across regions of the shell. While Lischka *et al*.^22^ observed the same patterns of regional calcification and related this to the shell repair, our results do not support the notion that the regional patterns were linked to repair processes potentially induced by shell dissolution. The inability to sustain calcification processes despite up-regulation of calcification-related genes^27, 30^ might thus be more specific to the loss of regional calcification capacity. The quality and content of food availability has been shown to offer some help against the negative effects of exposure history in some organisms (e.g., ref. 31). High calcification at low Ω_ar_ suggests that the site with the highest chl-a concentrations (e.g., Station 104) could provide individuals with a certain level of acclimatization capacity. The impact of chl-a on calcification is different from previous findings^21^, where the feeding efficiency observed as gut clearance rate did not increase at high CO_2_, but similar to observations of long-term feeding history, where chl-a positively affected energetic plasticity^32^. Shallowing slope of calcification capacity vs. Ω_ar_ conditions (Fig. 4) thus indicates acclimatization through enhanced compensation at Ω_ar_ < 1 conditions.

Calcification, growth, and survival all were observed to decline at Ω_ar_ ~1, suggesting that these processes are interconnected and occur at similar threshold value. Samples analyzed for both calcification and shell dissolution as related to exposure history (Supplementary Fig. S12) revealed a positive correlation between calcification and dissolution, with longer and more severe OA conditions triggering more extensive shell dissolution. This suggests that shell dissolution represents a physiological and energetic constraint that the organisms need to cope with in addition to calcification. Short-term exposure to severely undersaturated conditions can thus induce suppression of metabolism^27, 30, 32, 33^, which might temporarily serve as a short-term coping strategy to avoid energetically costly processes that could result in energy deficits. Specific environmental conditions can also impact survival capacity through energy-related constraints. For example, Arctic individuals of *L*. *helicina* with high lipid storage (on average 28% in the early stages^34, 35^) displayed higher survival rates^22^ (14% increase in mortality) in comparison with pteropods from the temporal regions with much lower lipid storage^36^ (2%; ref 37) and higher mortality (40% in this study).

OA exposure history, instead of food availability, is the crucial factor in determining individual survival success that could carry over to population-level responses. Reduced growth rates and developmental delays^22, 28^ imply that individuals might not attain specific lengths or sizes by the time of reproduction, thereby interfering with reproduction or imposing phenological changes in the timing of important life processes. Along with compromised early stages, the exposure of adults to severe OA conditions might carry over negative transgenerational effects mediated by maternal provisioning^29^ that could impose sublethal effects at the population level. We expect the largest effect on population growth rate to stem from proportional changes to the survival rates of juveniles and the subsequent first stage of sub-adults^38^, both of which could strongly influence regional pteropod abundance. Reduced pteropod abundances and biomass could have cascading effects through altered food-web dynamics and trophic relationships, and could have implications for biogeochemical cycling. The understanding of population-level effects could be improved with more specific energetic models that enhance linkages between vital biological processes, as well as through expanded monitoring of trends to allow for more direct estimation of pteropod status and potential ecological implications.

Overall, it appears to be difficult for *Limacina helicina* to maintain critical biological processes across the exposure regimes and spatial scales that comprise the pteropod’s natural habitat during the upwelling season. Our findings are comparable to those based on other species with similar vertical migration patterns, such as *Diacria quandridentata*, which exhibits reduced oxygen consumption and ammonia excretion under high-CO_2_ conditions in the natural environment. However, it differs from species that are naturally migrating into high CO_2_ zones^39^ (*Hyalocylis striata*, *Clio pyramidata*, *Cavolinia longirostris* and *Creseis virgule* ^40^) that might have developed adaptation strategies to cope with high CO_2_ exposure, at least in the short term.

The acceleration of OA in the CCE over the last few decades^4, 5, 8, 16^, has caused substantially reduced available habitat and remained uncompensated by acclimatization strategies or evolutionary adaptation. Considering that the life cycle of *L*. *helicina* in the CCE is completed after 1.2–1.5 years^33^, pteropods probably would have from 20 to 50 generations to respond to these changes. The potential for local adaptation could exist in those populations from the CCE that have not yet been sampled. However, lack of adaptive capacity to OA has also been demonstrated for other species, for example a non-native mussel species exposed to experimental treatments similar to those we used, despite vastly different evolutionary histories^34^. Renewed focus on the differentiation between populations and associated potential for acclimatization across a range of species will advance our understanding of ecosystem response to OA in the CCE.

## Conclusions

This study offers improved predictive capacity to bridge the current gap between responses of pteropods to OA at the individual and population levels as well as different degrees of climate change and associated ocean acidification. Exposure history influences the overall condition of individual pteropods, illustrating the importance of considering exposure history in the experimental design. Doing so will help reduce uncertainties in response variables and will provide more robust predictions of population-level responses to OA. Given the frequency of low Ω_ar_ conditions in the CCE, our observations suggest that acclimatization capacity limits may be small if any in pteropods from the coastal regions of the CCE. Our findings support the picture of a species increasingly constrained by the shifting physiochemistry of its ocean habitat, with a limited capacity to compensate for these changes. This conclusion appears to be corroborated by the observed decline in *Limacina helicina* in the northernmost part of the CCE^36^ during the last 20 years.

The absence of acclimatization capacity in these pteropods indicates that they can be used as sentinel organisms for OA monitoring, the context of which usually involves demonstrating population effects and a linkage of those effects with specific stressors in the field^41^. With adequate chemical and biological OA monitoring, potential population decline in this species should not go unnoticed in the CCE. Although the findings of this study focused on likely population effects on the US West Coast, these insights bear importance for other regions, particularly for the high-latitude environments where large pteropod abundance is ecologically important.

## Methods

For the 2013 West Coast Ocean Acidification cruise (WCOA2013; 1–28 August 2013) conductivity, temperature, depth and oxygen sensor profile data were collected along 10 cross-shelf transects accompanied by biological stations (Fig. 1) with accompanied vertical sections of temperature, salinity, nutrients, oxygen, calculated pCO_2_, pH, and calculated Ω_ar_ (Supplementary Fig. S1). At each station, water samples were collected in modified Niskin-type bottles, poisoned with HgCl_2_ and analyzed onboard the ship for dissolved inorganic carbon and total alkalinity (TA) (Fig. 1 and Supplementary Fig. S1). Due to the lack of chlorophyll shipboard observations, we used model output that included chl-a for stations 26, 28, 37, 38, 47, and 50 at three different depths: surface, 30 m, and 100 m. Chlorophyll distributions and concentrations were obtained from the J-SCOPE regional model^42^ and were either derived for the surface or were integrated over 30 m and 100 m depth over the period of 2 months, from 1 July to 31 August 2013 using the model tracking approach (see *Supplementary Information*). Chl-a fields were extracted from the model at the same sites observations were collected during that period. The modeled surface chlorophyll is poorly correlated with the sparse observations from the same time period (R^2^ = 0.35) but performs well for other fields (e.g., oxygen, temperature, pH, Ω, nutrients). We used correlation analysis, principal components analysis, cluster analysis, and modeling results to describe comparisons of water chemistry within and among different sites. Pteropods were collected at the biological subset of stations (Fig. 1) using 200 µm mesh Bongo nets, with integrated sampling over the upper 100 m. Out of the net tows, *Limacina helicina* individuals were selected for the experimental use. All the pteropods were examined for mechanical damage; only intact and actively swimming individuals were placed in the experimental conditions, and were considered for the analyses at the end of the experiments.

We conducted two different types of experiments to determine: 1) calcification, and 2) survival under high CO_2_ conditions among the pteropods collected from different stations. For the calcification study, we examined the responses of 96 pteropods from 12 different stations (Supplementary Table S3). Calcification was quantified using calcein staining over a 24-hour period in ambient water from which the pteropods originated. Calcein dye stained the shell^20, 22^ and under UV-light produced fluorescence at the sites of active calcification in the shell. Although we measured three response variables for calcification (proportional glow, mean intensity, and growing edge intensity; see *Supplementary Information*), we used only proportional glow (i.e., proportion of shell surface area that was fluorescing) because it was a good predictor of mean intensity and edge intensity (Supplementary Fig. S5). In the process of standardization (Supplementary Fig. S3), measurements of glowing shell area were divided by the total shell area to normalize to individual size.

We used a two-step analysis to determine whether calcification varied in relation to local water chemistry. First, we used generalized linear models (GLMs) to establish whether proportional glow varied among pteropods collected from different stations (see *Supplementary Information*). Next we used the predicted means as the response variable for a second round of GLMs (with variance as weights) to determine whether environmental parameters explained the variation in proportional glow among stations. We included Ω_ar_ as the primary predictor variable because it is most likely to directly impact calcification. We then regressed TA, temperature, and pH against Ω_ar_ and used their residuals as additional predictors (see *Supplementary Information*). In both cases, we fit several models (step one included only a null model and a station model), including the null model, and compared Akaike Information Criteria (AICc) values to pick the best-fit model^25^. This approach allowed us to distinguish between three possibilities: 1) calcification did not vary among stations, 2) calcification varied among stations but was not related to environmental conditions, and 3) calcification varied among stations and was related to environmental conditions.

To determine whether survival probability under high CO_2_ conditions was related to the carbonate chemistry of the water of origin, we placed captured pteropods in shipboard flow-through aquaria with pre-acclimatized water to targeted CO_2_ levels of ~400, 800, or 1200 µatm. We maintained pteropods (altogether 570 individuals; Supplementary Table S7) from 10 different stations under treatment conditions for one week to investigate their probability of survival at each treatment level. A two-step statistical analysis similar to that described above (GLM, logit-link, and binomial error) was conducted to estimate survival, using experimental Ω_ar_ and station identity as fixed categorical factors in the first step (see *Supplementary Information* for details).

In addition, we have related calcification and chlorophyll concentrations, i.e., food availability, to biological responses, both for calcification and survival probability. We ran a similar sub-analysis with Ω_ar_ and chl-a concentration (only available for stations 26, 28, 37, 38, 47, and 50) at three depths: surface, 30 m, and 100 m. Due to lack of data (only 6 data points overlapping between chl-a concentration and calcification/survival data), it was not possible to run the model with combined Ω_ar_ and chl-a (see Supplementary Table S12). To estimate conditions and duration of exposure history to low Ω_ar_, we used a high-resolution hindcast model simulation of 2013 ocean conditions in the US Pacific Northwest^42^ to track particles released along transects between 44.5 and 48° N, coincident with sampling locations. We utilized hourly values of model velocities (which included tides) for the particle tracking and daily average model fields of aragonite undersaturation to determine the range of saturation conditions the particle tracks experienced during their recent (5–6 weeks) history. Pteropod-like diel vertical migration between 10 and 100 m depth was imposed on the particles, which influenced their exposure; in particular, the particles experienced more exposure to low Ω_ar_ at depth. Particles were released (*in silico*) along the sampled transects on 1 August 2013 and were tracked for one month backward in time (to 1 July 2013) and one month forward in time (to 31 August 2013) to provide exposure history for all particles throughout the entire cruise duration (1–31 August 2013). To track floats backward in time, we interpolated the velocity field to the location of each numerical float, and used those values to calculate where the floats were located at the *previous* time step. As a simple example using the Euler method: if the velocity field indicates a flow of *u* to the east at the float location at time *t*, forward tracking with the Euler method would translate the float u*dt (e.g., to the east for *u* > 0), whereas backward tracking translates it −u*dt (e.g., to the west for *u* > 0). Subsequently, for backward particle tracking, we sample the velocity field at time *t-dt* and repeat the process. In this manner, we work successively backward in time to calculate the likely path a float took to its present location. These and related concepts for particle tracking are discussed by Brickman *et al*.^43^. A total of 2000 particles were spread across the study area. For specific calculations of exposure history applicable to each pteropod field sample, we utilized only those particles that were within 25 km cross-shelf and 125 km alongshore distance of the pteropod sample location on the sampling date, and calculated the mean exposure history of that subsample of particles over the previous 30 days. As our metric of exposure history, we calculated a severity index *S* (*sensu* ref. 5), which defines exposure as a combined effect of duration and magnitude of undersaturation, termed “undersaturation-days”:

1 $$S=I\,\ast \,D$$

where *D* is defined as the total duration of time the water is undersaturated over some time

2 $$I=\frac{\sum _{j=1}^{N}({\int }_{t=0}^{{t}_{j}}\{T-{{\rm{\Omega }}}_{a}(t)\}dt/{t}_{j})}{N}$$

where *T* is a specific threshold value, ${\Omega }_{a}$ is the aragonite saturation state, *N* is the number of events over time interval *d*, and *t*_*j*_ is the length of each event. The value for *T* was assumed to be 1 for this study.

### Data Availability

The data reported in this paper are tabulated in the Supplementary Information and will be archived at the National Centers for Environmental Information.

## Electronic supplementary material

Supplementary Material
