# Supplementary material for: Exposure history determines pteropod vulnerability to ocean acidification along the US West Coast
Source: Sci Rep. 2017 Jul 3;7:4526. doi: 10.1038/s41598-017-03934-z (PMC5495755; doi:10.1038/s41598-017-03934-z)
Supplement: Supplementary file 1 — Supplementary Material [file 41598_2017_3934_MOESM1_ESM.pdf]

Supplementary Information

- **Title Page with the Title of the Manuscript and the Author List**
- **Supplementary Figures S1–S6**
- **Supplementary Tables S1–S11**
- **Supplementary Information Text and References**

Title: Exposure history determines pteropod vulnerability to ocean acidification along the US West Coast

Authors: N. Bednaršek[1,*,#], R.A. Feely[1], N. Tolimieri[2], A.J. Hermann[1,3], S.A. Siedlecki[3], G.G. Waldbusser[4], P. McElhany[2], S.R. Alin[1], T. Klinger[5], B. Moore-Maley[6], H.O. Pörtner[7]

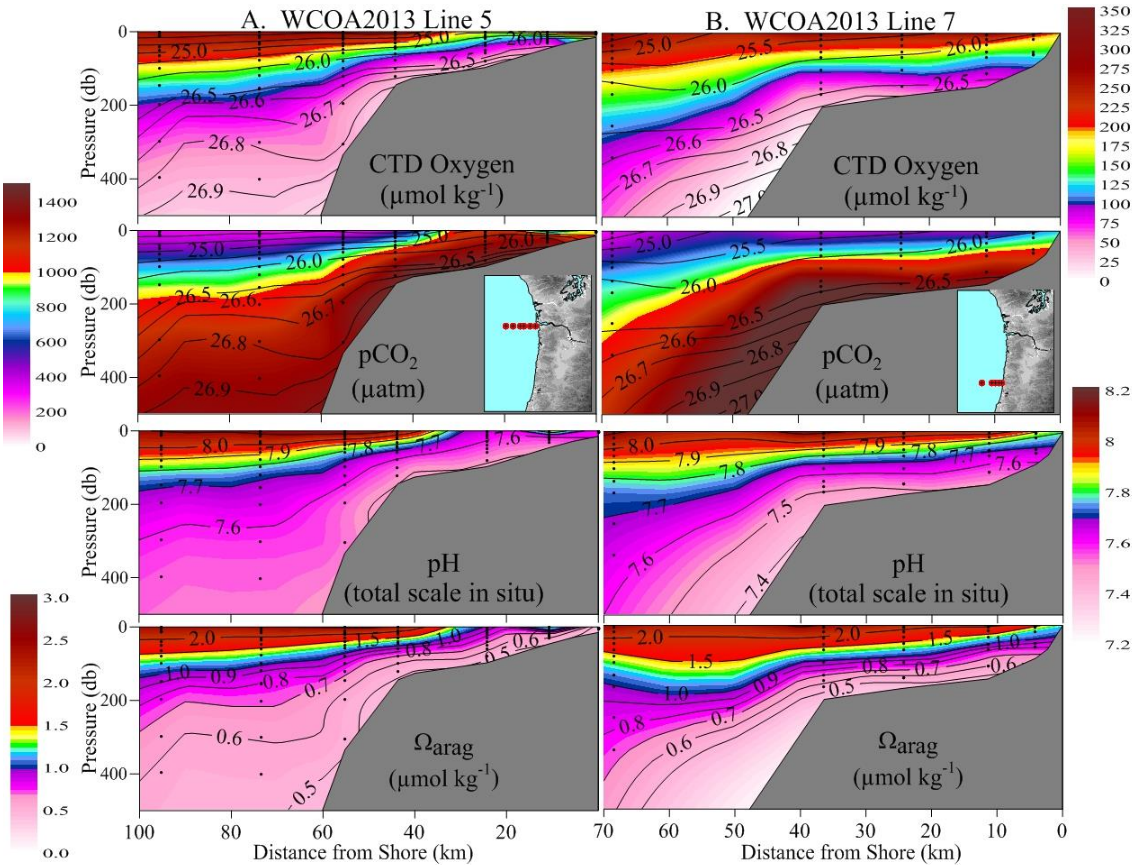

Supplementary Figure S1: Vertical sections of oxygen, pCO_2, pH, and Ω_{ar} off the Columbia River (**a**) and near Heceta Bank (**b**) on the Oregon Continental Shelf. Black dots indicate measurement locations and the contour lines in **a** and **b** show the potential density in kg m^{-3}.

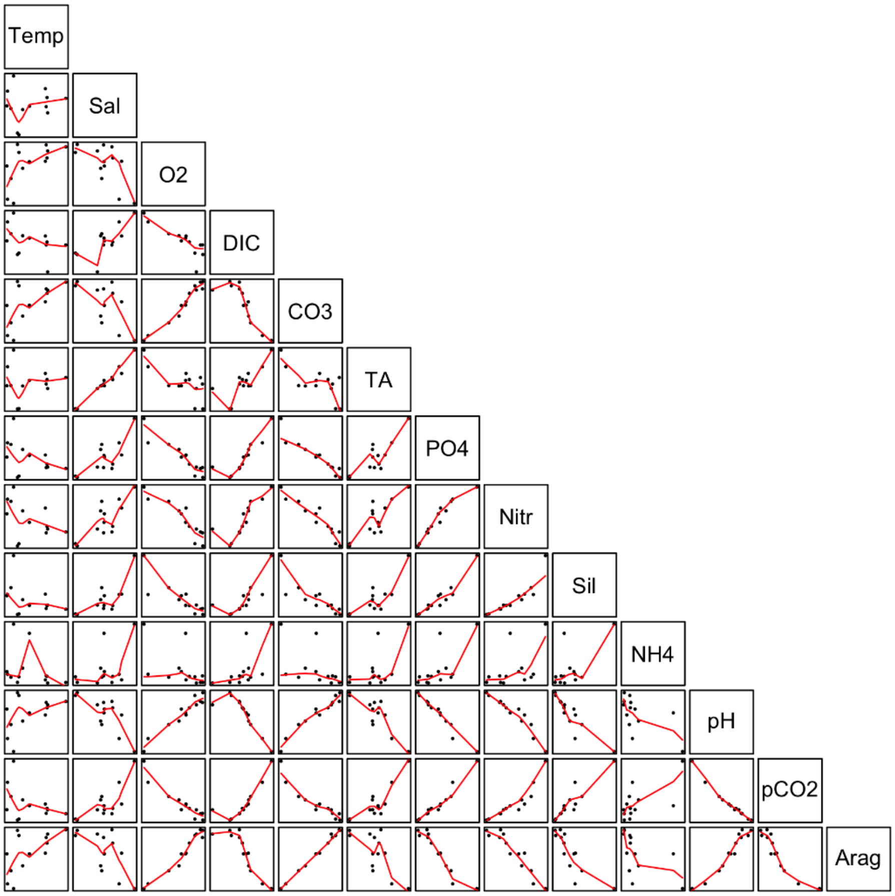

Supplementary Figure S2: Correlations among environmental parameters among sites.

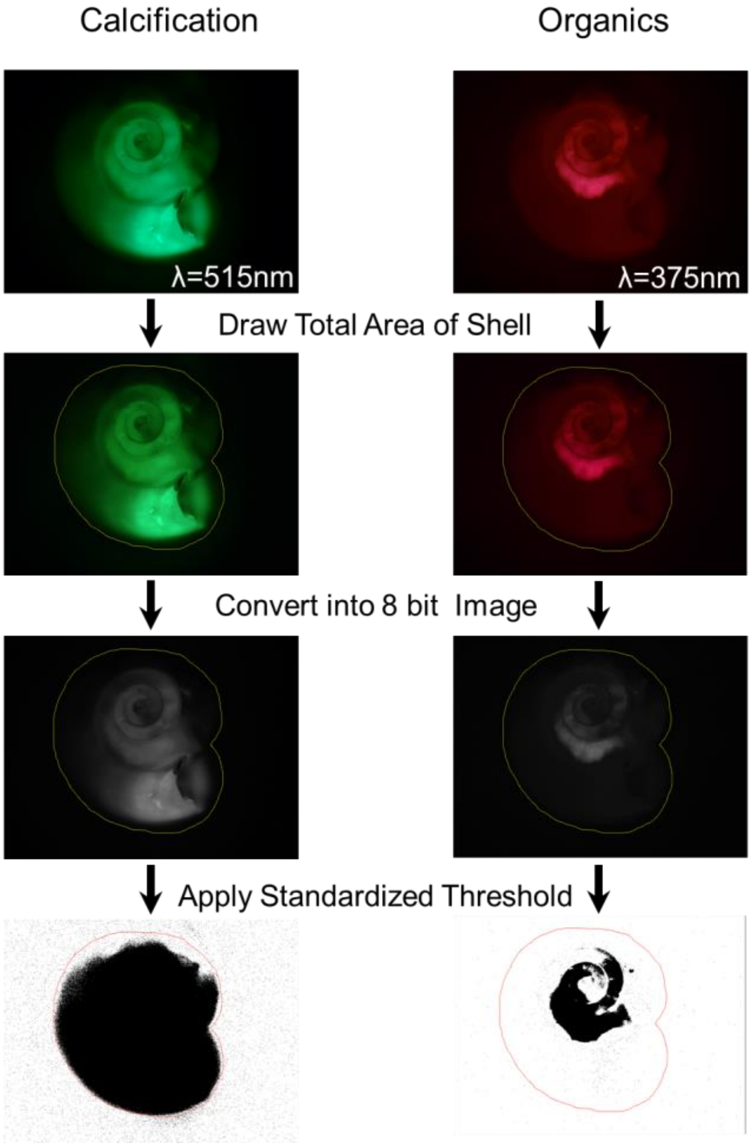

Supplementary Figure S3: Method of determining total calcification area and intensity (left top to bottom) and differentiating from the proportion of organic material (right top to bottom) using a standardized threshold.

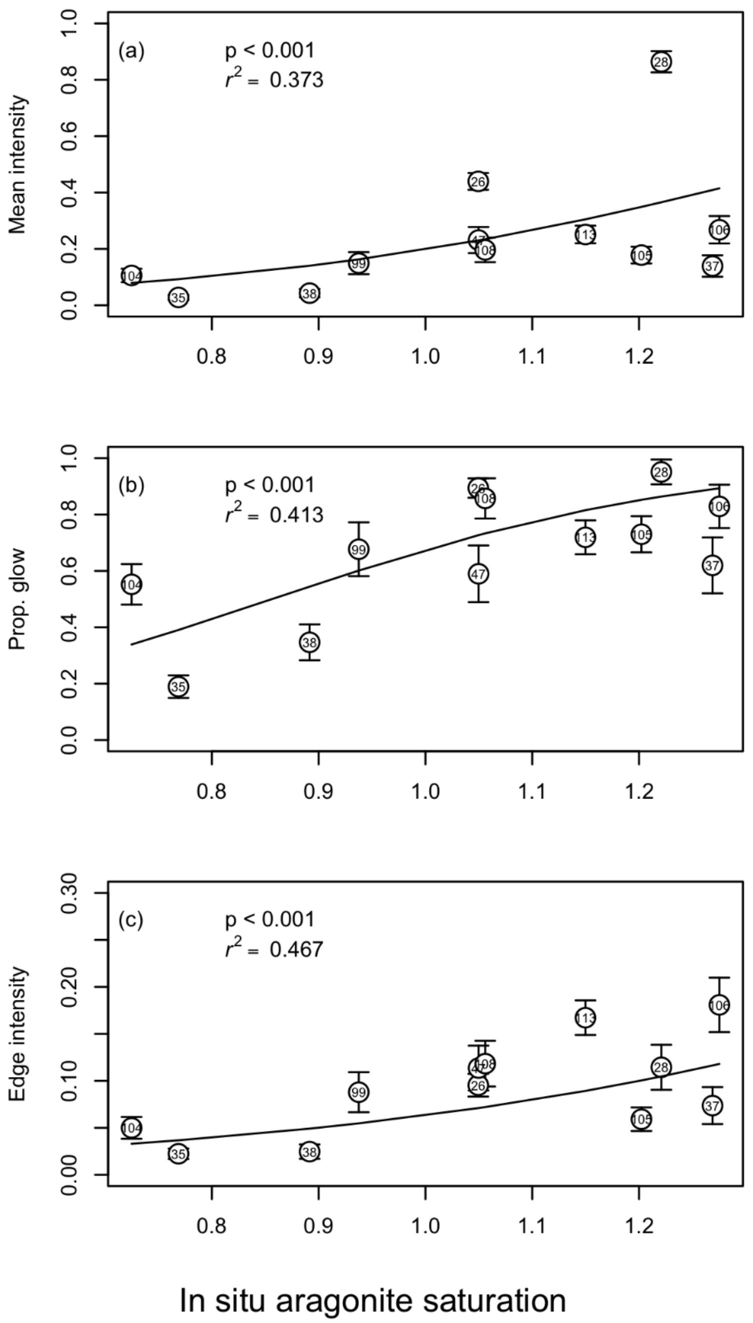

Supplementary Figure S4: Three different calcification metrics: (**a**) mean shell intensity, (**b**) proportional glow, and (**c**) edge intensity, varying in the intensity, extent, and location of fluorescence at different omega saturation states of the stations (station numbers appear inside circles).

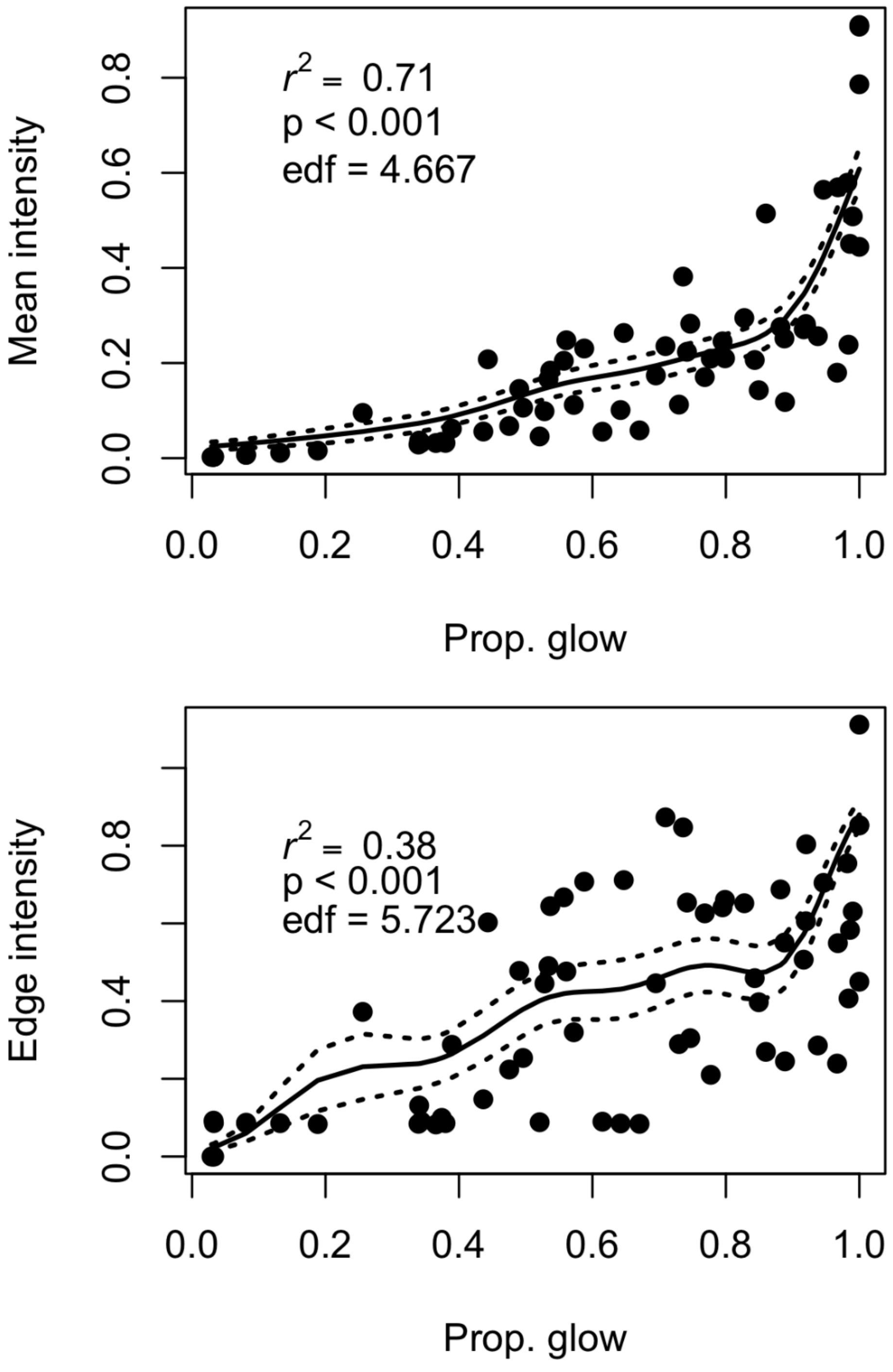

Supplementary Figure S5: Results of generalized additive models (beta error distribution and logit-link) examining the relationship between proportional glow and (**a**) mean intensity, and (**b**) edge intensity, determined by the standardized method (based on Supplementary Fig. S3).

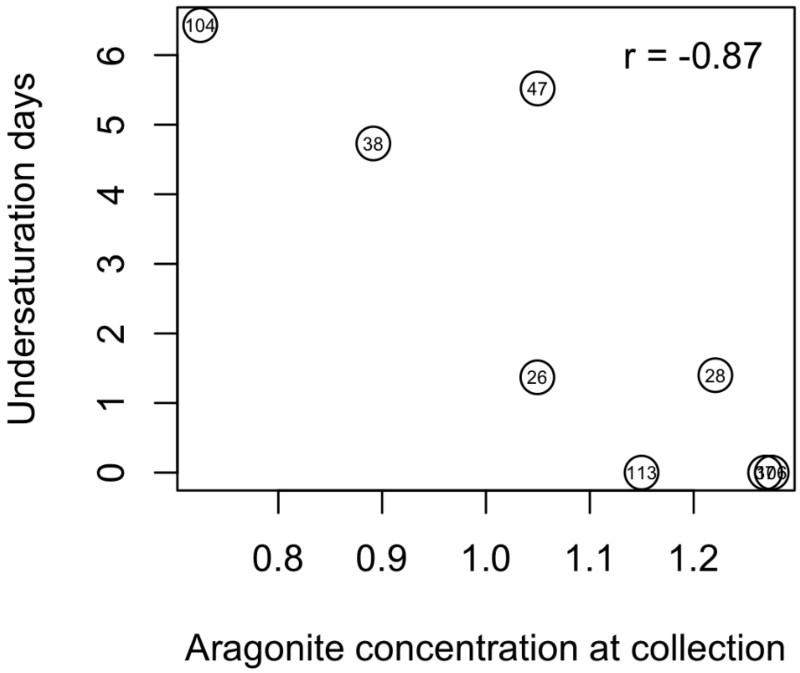

Supplementary Figure S6: Correlation between observed in situ aragonite saturation state (at the origin of pteropod collection) and model output (undersaturation days).

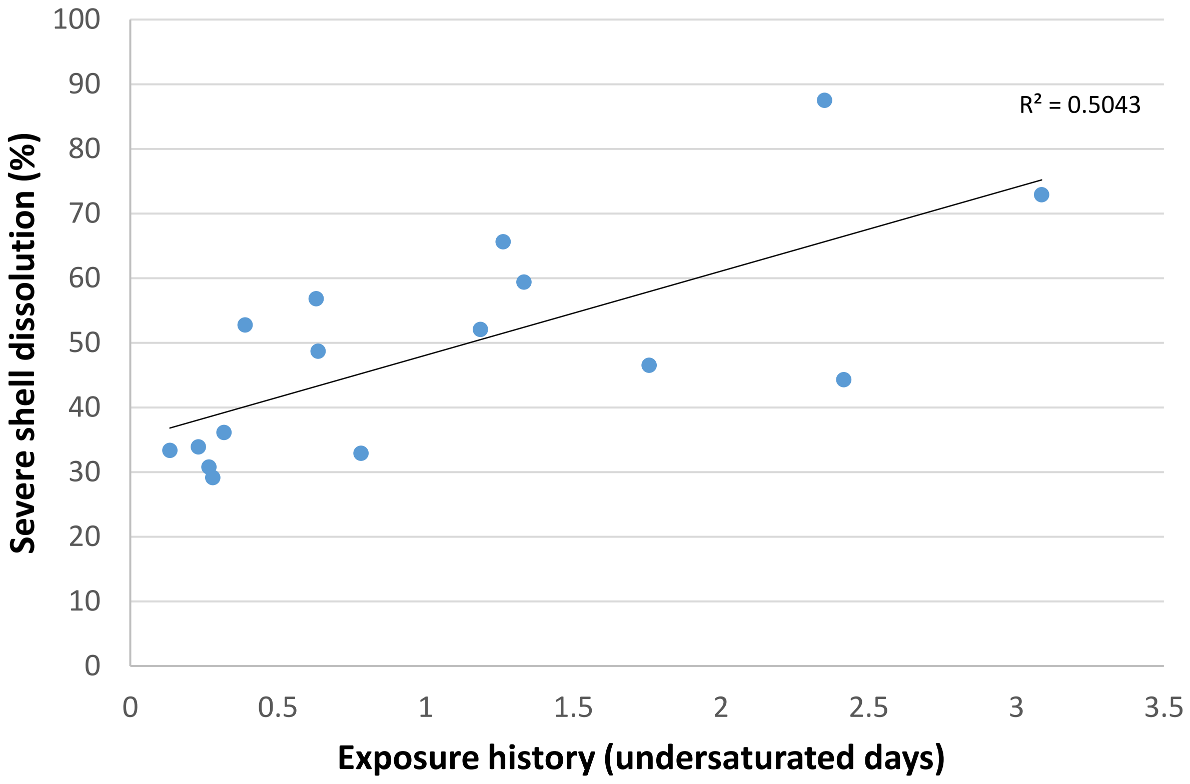

Supplementary Figure S7: The impact of exposure history on severe shell dissolution (Type II and Type III) with correlation of R^2= 0.504. The results on severe shell dissolution are from Feely et al. (2016). Method section provides an explanation on how the saturation history was derived.

Supplementary Table S1: Pearson’s correlations among environmental variables among sampling sites.

Salinity	0.17											
O_2	0.59	-0.63										
DIC	-0.34	0.83	-0.94									
CO_3^{2-}	0.6	-0.62	0.97	-0.94								
TA	0.07	0.98	-0.72	0.9	-0.7							
PO_4	-0.42	0.75	-0.92	0.97	-0.95	0.83						
Nitrate	-0.39	0.78	-0.9	0.95	-0.94	0.81	0.96					
Silicate	-0.38	0.73	-0.84	0.89	-0.84	0.78	0.95	0.89				
NH_4	-0.26	0.43	-0.52	0.6	-0.54	0.55	0.66	0.47	0.63			
pH	0.42	-0.75	0.96	-0.99	0.97	-0.83	-0.98	-0.95	-0.89	-0.59		
pCO_2	-0.4	0.75	-0.94	0.98	-0.93	0.84	0.97	0.9	0.92	0.64	-0.98	
Ω_{ar}	0.59	-0.62	0.97	-0.94	1	-0.7	-0.95	-0.94	-0.83	-0.54	0.97	-0.92
	Temp	Salinity	O_2	DIC	CO_3^{2-}	TA	PO_4	Nitrate	Silicate	NH_4	pH	pCO_2

Supplementary Table S2: Principal component (PC) loadings examining variation among stations in environmental characteristics. Eigenvalues for the first two components were 2.95 and 1.20, respectively. The first two axes explain 79% and 13% of the variation, respectively.

	PC 1	PC 2
Temperature	-0.146	0.705
Salinity	0.244	0.485
O_2	-0.299	0.164
DIC	0.280	-0.001
CO_3^{2-}	-0.301	0.174
TA	0.265	0.420
PO_4	0.310	0.006
Nitrate	0.299	0.026
Silicate	0.289	0.042
NH_4	0.205	0.004
pH	-0.310	0.004
pCO_2	0.305	0.024
Ω_{ar}	-0.301	0.178

Supplementary Table S3: **Carbonate chemistry conditions in the natural environment.** Results represent interpolated values for the upper 100 m water depth for each parameter. Phytoplankton concentrations (max) were taken at 30 m depth. Second column indicates cluster groups. The final column is the number of individuals used calcification experiment with seawater chemistry from the particular station. N/A indicates that phytoplankton was not taken at that station. Additional information on the cruise data can be found at http://cdiac.ornl.gov/ftp/oceans/WCOA2013/ .

Station	Date	Cluster Group	T (°C)	Sal	O_2 *(µmol/kg)*	DIC *(µmol/kg)*	CO_3^{2-} *(µmol/kg)*	TAlk *(µmol/kg)*	PO_4 *(µmol/kg)*	pH	Chl conc. (max)	pCO_2	Ave Ω_{ar}	# ind.
**26**	08-06-13	2	7.4	33.3	163	2147.90	74.44	2225.60	1.86	7.80	N/A	720	1.05	10
**28**	08-07-13	3	7.9	32.9	189	2105.17	85.36	2202.30	1.54	7.87	N/A	604	1.22	3
**35**	08-08-13	1	7.5	33.6	98	2214.42	57.73	2245.00	2.11	7.65	2.5	1062	0.77	12
**37**	08-09-13	3	7.8	32.9	204	2099.97	87.73	2202.04	1.52	7.89	0.7	591	1.27	3
**38**	08-08-13	2	7.6	33.3	138	2173.05	64.83	2225.75	2.08	7.72	2.1	885	0.89	7
**47**	08-10-13	2	8.2	33.33	171	2157.66	74.62	2231.08	1.89	7.78	N/A	770	1.05	3
**99**	08-22--13	2	8.0	33.3	158	2165.92	68.56	2225.37	1.99	7.74	N/A	843	0.94	3
**104**	08-22-13	1	7.7	33.8	91	2245.82	55.05	2261.97	2.52	7.58	N/A	1326	0.72	6
**105**	08-23-13	4	8.8	33.3	203	2133.79	83.64	2227.38	1.68	7.84	N/A	675	1.20	6
**106**	08-23-13	4	9.5	33.5	199	2133.49	87.41	2233.99	1.67	7.84	N/A	670	1.28	3
**108**	08-23-13	2	8.8	33.6	172	2164.95	76.49	2238.20	1.90	7.77	N/A	791	1.06	3
**113**	08-24-13	4	8.8	33.5	178	2144.09	81.17	2231.91	1.74	7.81	N/A	737	1.15	7

Supplementary Table S4: Model selection parameters for models predicting calcification (proportional glow) of pteropods from multiple stations. Ω_{ar} = aragonite saturation state, TA = total alkalinity, temp = temperature °C. TA, temp, and pH are all residuals of those terms regressed against Ω_{ar} to control for multicollinearity. Selection was based on AICc weights (w_i). Models were generalized linear models with logit link and beta error distribution AICc = Akiake's Information Criterion for small sample sizes. res.Temp and res.TA were the residuals temperature (°C) and TA regressed against Ω_{ar} to control for multicollinearity.

**Model**	**AICc**	**Delta AICc**	**Weight**
Null	-479.90	576.8	0
Ω_{ar}	-989.35	67.4	0
Ω_{ar} + res.TA	-996.84	59.9	0
Ω_{ar} + res.temp	-1040.43	16.3	0
Ω_{ar} + res.pH	-1039.71	17.03	0
Ω_{ar} + res.TA + res.temp	-1054.80	1.93	0.27
Ω_{ar} + res.TA + res.pH	-1056.73	0	0.72
Ω_{ar} + res.temp + res.pH	-1034.50	22.4	0
Ω_{ar} + res.TA + res.temp + res.pH	-1048.54	8.19	0.01

Supplementary Table S5: Model coefficients for the best-fit model in Supplementary Table S2 to predict proportional glow: generalized linear model with logit-link and beta error distribution. Ω_{ar} = aragonite saturation coefficient; Temp = residuals of temperature (°C) versus Ω_{ar}; pH= residuals of pH versus Ω_{ar}; TA = residuals of total alkalinity versus Ω_{ar}; S.E. = standard error of the estimate.

Proportional Glow	Coefficient	S.E.	p-value
Intercept	-4.864	0.191	< 0.001
Ω_{ar}	5.529	0.181	< 0.001
pH (residuals)	0.023	0.095	< 0.001
TA (residuals)	-0.732	0.005	< 0.001

Supplementary Table S6: Calcification data for each individual with measurements of three different calcification metrics: mean intensity, proportion glow, and edge intensity (provided as raw and as intensity area). The data accompany that of Supplementary Figure S4. Complementary environmental data for each station at which individual pteropods were taken can be found in Supplementary Table S3 (which provides the number of organisms in the last column).

station	mean intensity	proportion glow	edge intensity (raw data)	edge intensity (area)
26	51.44	85.99	26.94	5.84
26	27.09	91.66	50.67	7.70
26	17.05	76.84	62.59	7.98
26	38.18	73.59	84.71	12.67
26	56.44	94.64	70.44	10.77
26	44.44	100.00	44.99	5.22
26	56.96	96.78	54.92	7.76
26	45.07	98.60	58.33	7.50
26	57.91	98.20	75.46	9.69
26	50.85	99.02	63.05	9.03
47	18.40	53.68	64.45	13.42
47	24.78	56.09	47.62	5.38
47	23.55	70.95	87.30	13.52
105	28.28	74.66	30.47	2.52
105	14.28	84.93	39.71	3.60
105	11.81	88.86	24.49	2.90
105	20.93	77.73	21.02	2.19
105	11.19	57.21	31.98	8.50
105	17.39	69.51	44.59	12.72
38	5.56	61.50	8.97	1.47
38	3.43	37.42	9.94	1.19
38	4.56	52.09	8.82	1.30
38	0.69	8.09	8.57	0.28
38	0.29	3.22	9.23	0.06
38	10.12	64.22	8.50	0.99
38	5.59	43.65	14.73	2.48
104	20.47	55.71	66.71	11.39
104	6.19	38.94	28.76	3.67
104	21.00	79.87	66.06	8.07
104	3.20	37.98	8.59	0.84

104	5.84	67.06	8.41	0.50
104	9.86	52.82	44.54	5.14
28	90.75	100.00	111.16	12.59
28	78.63	100.00	85.27	9.16
28	91.08	100.00	85.23	9.06
37	2.86	33.93	8.43	0.95
37	25.17	88.77	55.07	7.95
37	23.06	58.77	70.73	17.65
113	29.47	82.76	65.19	20.90
113	28.20	91.99	60.55	8.93
113	22.36	74.17	65.36	17.60
113	20.78	44.32	60.27	19.37
113	26.32	64.70	71.14	18.82
113	27.43	92.04	80.36	13.23
113	16.64	53.42	49.01	13.51
35	0.30	3.31	0.00	0.00
35	0.26	2.91	0.00	0.00
35	9.52	25.57	37.22	9.52
35	3.69	34.03	13.14	1.34
35	0.29	3.26	8.60	0.07
35	3.18	36.55	8.25	0.30
35	6.76	47.53	22.39	4.09
35	3.19	34.32	9.06	0.85
35	0.69	8.11	8.71	0.33
35	1.11	13.20	8.59	0.14
35	0.28	3.16	0.00	0.00
35	1.58	18.83	8.35	0.93
108	14.55	49.03	47.77	12.09
108	23.86	98.38	40.68	16.09
108	17.97	96.67	23.92	5.77
99	10.60	49.60	25.36	4.04
99	20.64	84.32	45.91	15.25
99	11.26	72.97	28.95	6.14
106	27.58	88.18	68.75	11.02
106	24.56	79.50	64.14	17.42
106	25.65	93.78	28.58	24.71

Supplementary Table S7: Raw pteropod data from the original water placed in the experimental conditions with pre-acclimatized water at 400, 800, and 1200 uatm. Two sets of high CO_2 experimental treatment are identified (treatments 1 and 2 conducted in the first and second part of the cruise, respectively); each lasting for 1 week in which pCO_2 was continuously bubbled. Within each treatment, the water was supplied to 3 crates with 6 bottles inside, between which the water was constantly recirculating. pH was measured twice daily, based on which standard deviation was calculated. CO_2 pressure in the gas bottle was estimated with the precision of ±5%. TA, TC, and omega were calculated using CO2SYS. Carbonate chemistry is provided as the range of conditions to which the organisms were exposed. Sample size as well as the range of pH, pCO_2, TC, TA and Ωar conditions for each high CO2 survival experiment is provided.

station	exp.	Treat	pCO2	bottle #	N	live	dead	%surv.	TP	Tsi	T	pH	pCO2	TA	TC	Ω
									(umol/kg)	*(umol/kg)*	*(° C)*		*(uatm)*	*(umol/kg)*	*(umol/kg)*	
26	1C	1	400	1	11	6	5	0.55	0.31	1.47	10	8.04-8.10	380-420	2110-2728	1964-2512	1.8-2.6
26	2C	1	800	7	18	6	12	0.33	0.31	1.47	10	7.74-7.78	760-840	1986-2418	1930-2342	0.88-1.18
26	3C	1	1200	13	18	6	12	0.33	0.31	1.47	10	7.61-7.69	1140-1260	2159-2895	2137-2845	0.72-1.16
28	1B	1	400	2	41	23	18	0.56	0.31	1.47	10	8.04-8.10	380-420	2110-2728	1964-2512	1.8-2.6
28	2B	1	800	8	40	13	27	0.33	0.31	1.47	10	7.74-7.78	760-840	1986-2418	1930-2342	0.88-1.18
28	3B	1	1200	14	38	14	24	0.37	0.31	1.47	10	7.61-7.69	1140-1260	2159-2895	2137-2845	0.72-1.16
47	1A	1	400	3	16	7	9	0.44	0.31	1.47	10	8.04-8.10	380-420	2110-2728	1964-2512	1.8-2.6
47	2A	1	800	9	14	2	12	0.14	0.31	1.47	10	7.74-7.78	760-840	1986-2418	1930-2342	0.88-1.18
47	3A	1	1200	15	25	4	21	0.16	0.31	1.47	10	7.61-7.69	1140-1260	2159-2895	2137-2845	0.72-1.16
37	1D	1	400	4	15	12	3	0.80	0.31	1.47	10	8.04-8.10	380-420	2110-2728	1964-2512	1.8-2.6
37	2D	1	800	10	15	6	9	0.40	0.31	1.47	10	7.74-7.78	760-840	1986-2418	1930-2342	0.88-1.18
37	3D	1	1200	16	15	7	8	0.47	0.31	1.47	10	7.61-7.69	1140-1260	2159-2895	2137-2845	0.72-1.16
38	1E	1	400	5	11	6	5	0.55	0.31	1.47	10	8.04-8.10	380-420	2110-2728	1964-2512	1.8-2.6
38	2E	1	800	11	14	4	10	0.29	0.31	1.47	10	7.74-7.78	760-840	1986-2418	1930-2342	0.88-1.18
38	3E	1	1200	17	22	2	20	0.09	0.31	1.47	10	7.61-7.69	1140-1260	2159-2895	2137-2845	0.72-1.16
50	1F	1	400	6	26	16	10	0.62	0.31	1.47	10	8.04-8.10	380-420	2110-2728	1964-2512	1.8-2.6
50	2F	1	800	12	24	14	10	0.58	0.31	1.47	10	7.74-7.78	760-840	1986-2418	1930-2342	0.88-1.18
50	3F	1	1200	18	18	11	7	0.61	0.31	1.47	10	7.61-7.69	1140-1260	2159-2895	2137-2845	0.72-1.16
108	1B	2	400	1	10	4	6	0.40	1.14	12.42	10	8.03-8.07	380-420	2085-2545	1927-2347	1.72-2.28
108	2B	2	800	7	21	9	12	0.43	1.14	12.42	10	7.73-7.79	760-840	1947-2489	1890-2406	0.86-1.26
108	3B	2	1200	13	32	10	22	0.31	1.14	12.42	10	7.58-7.64	1140-1260	2237-2341	2222-2307	0.72-0.86
106	1A	2	400	2	39	28	11	0.72	1.14	12.42	10	8.03-8.07	380-420	2085-2545	1927-2347	1.72-2.28
106	2A	2	800	8	49	30	19	0.61	1.14	12.42	10	7.73-7.79	760-840	1947-2489	1890-2406	0.86-1.26
106	3A	2	1200	14	28	17	11	0.61	1.14	12.42	10	7.58-7.64	1140-1260	2237-2341	2222-2307	0.72-0.86
105	1E	2	400	3	22	12	10	0.55	1.14	12.42	10	8.03-8.07	380-420	2085-2545	1927-2347	1.72-2.28
105	2E	2	800	9	29	12	17	0.41	1.14	12.42	10	7.73-7.79	760-840	1947-2489	1890-2406	0.86-1.26
105	3E	2	1200	15	32	12	20	0.38	1.14	12.42	10	7.58-7.64	1140-1260	2237-2341	2222-2307	0.72-0.86
113	1D	2	400	4	20	16	4	0.80	1.14	12.42	10	8.03-8.07	380-420	2085-2545	1927-2347	1.72-2.28
113	2D	2	800	10	40	23	17	0.58	1.14	12.42	10	7.73-7.79	760-840	1947-2489	1890-2406	0.86-1.26
113	3D	2	1200	16	34	21	13	0.62	1.14	12.42	10	7.58-7.64	1140-1260	2237-2341	2222-2307	0.72-0.86

Supplementary Table S8: Survival results for each treatment (upper) and for the each station at 1200 ppm (lower), with mean survival at each station (column 'mean') and on the cluster level (i.e. orders in the last category). Included also the number of initial organisms in the treatment (from Supplementary Table S7). The raw data are presented in Supplementary Table S7.

pCO_2	coeff	se	mean	upper	lower	order
1200	-0.46	0.27	0.38	0.46	0.33	3
400	0.51	0.277	0.62	0.7	0.56	1
800	-0.22	0.26	0.45	0.51	0.38	2

<table>
<tr><th>station</th><th>coeff</th><th># ind at the start</th><th>se</th><th>mean</th><th>upper</th><th>lower</th><th>order</th><th>means by cluster</th></tr>
<tr><td>28</td><td>-0.33</td><td>20</td><td>0.22</td><td>0.42</td><td>0.47</td><td>0.37</td><td>1</td><td rowspan="2">0.49</td></tr>
<tr><td>37</td><td>0.23</td><td>16</td><td>0.33</td><td>0.56</td><td>0.64</td><td>0.48</td><td>1</td></tr>
<tr><td>50</td><td>0.44</td><td>18</td><td>0.28</td><td>0.61</td><td>0.67</td><td>0.54</td><td>2</td><td rowspan="4">0.58</td></tr>
<tr><td>106</td><td>0.63</td><td>22</td><td>0.23</td><td>0.65</td><td>0.70</td><td>0.60</td><td>2</td></tr>
<tr><td>105</td><td>-0.27</td><td>22</td><td>0.25</td><td>0.43</td><td>0.49</td><td>0.37</td><td>2</td></tr>
<tr><td>113</td><td>0.59</td><td>19</td><td>0.24</td><td>0.64</td><td>0.70</td><td>0.58</td><td>2</td></tr>
<tr><td>47</td><td>-1.21</td><td>22</td><td>0.34</td><td>0.23</td><td>0.30</td><td>0.18</td><td>3</td><td rowspan="4">0.31</td></tr>
<tr><td>108</td><td>-0.56</td><td>19</td><td>0.28</td><td>0.36</td><td>0.43</td><td>0.30</td><td>3</td></tr>
<tr><td>26</td><td>-0.49</td><td>21</td><td>0.32</td><td>0.38</td><td>0.46</td><td>0.31</td><td>3</td></tr>
<tr><td>38</td><td>-1.10</td><td>18</td><td>0.36</td><td>0.25</td><td>0.32</td><td>0.19</td><td>3</td></tr>
</table>

Supplementary Table S9: Model selection parameters for pteropod survival among laboratory pCO_2 treatments and stations where the pteropods were collected. The best-fit model (in bold) included laboratory pCO_2 and Station as factors but not their interaction.

Selection parameters for models predicting survival of pteropods from multiple stations. Ω = aragonite saturation state, TA = total alkalinity, temp = temperature °C. TA, temp, and pH are all residuals of those terms regressed against Ω to control for multicollinearity. Bold indicates the best-fit model.

Model	AICc	delta AICc
pCO_2 + Station + pCO_2 x Station	991.3	27.0
**pCO_2 + Station**	**964.3**	**0.0**
pCO_2	1002.5	38.2
Station	981.5	17.2
Null	1022.4	58.1

Supplementary Table S10: Coefficients from the best-fit model to explain variation in pteropod survival among laboratory pCO_2 treatments and stations where the pteropods were collected. The model included laboratory pCO_2 and Station as factors but not their interaction.

Coefficients:	Estimate	S.E.	z-value	p
(Intercept)	-0.515	0.247	-2.082	0.037
1200 pCO_2	0.832	0.2	4.168	<0.001
800 pCO_2	0.065	0.185	0.351	0.725
Station 106	0.834	0.3	2.781	0.005
Station 108	-0.201	0.348	-0.578	0.564
Station 113	0.895	0.313	2.858	0.004
Station 26	-0.192	0.379	-0.507	0.612
Station 28	-0.125	0.294	-0.425	0.671
Station 37	0.45	0.379	1.186	0.236
Station 38	-0.797	0.407	-1.959	0.05
Station 47	-0.952	0.393	-2.42	0.016
Station 50	0.609	0.338	1.799	0.072

Supplementary Table S11: Selection parameters for models predicting survival of pteropods from multiple stations. Ω = aragonite saturation state, TA = total alkalinity, temp = temperature °C. TA, temp, and pH are all residuals of those terms regressed against Ω to control for multicollinearity. Bold indicates the best-fit model.

Model	AICc	Delta AICc	Weight
Null	-5.2	4.82	0.06
**Ω**	**-10.02**	**0**	**0.67**
Ω + TA	-5.77	4.25	0.08
Ω + temp	-5.53	4.49	0.07
Ω + pH	-6.41	3.61	0.11
Ω + TA + temp	3.23	13.24	0
Ω + TA + pH	2.57	12.59	0
Ω + temp + pH	2.39	12.4	0
Ω + TA + temp + pH	16.93	26.95	0

Supplementary Table S12: Model selection parameters via AIC and correlation coefficient (R^2) for pteropod calcification and survival probability (upper and lower table, respectively), including Ω_{ar} and chla concentration but not their interaction. Bold indicates the best-fit model. N indicates sample size at the available stations with overlap between chla and experimental data.

**AIC (calcification)**	**100 m**	**30 m**	**Surface**
Null	-263	-263	-274
Ω_{ar}	**-696**	**-696**	**-752**
Chla conc	-450	-424	-307
Ω_{ar} + Chla conc	NA	NA	NA
N	6	6	7
**R^2**			
Null	NA	NA	NA
Ω_{ar}	**0.44**	**0.44**	**0.42**
Chla conc	0.11	0.12	0.004
Ω_{ar} + Chla conc	NA	NA	NA

**AIC (survival)**	**100 m**	**30 m**	**Surface**
Null	-3.96	3.96	-3.96
Ω_{ar}	**-9**	**-9**	**-9**
Chla conc	-2.69	-2.93	-3.79
Ω_{ar} + Chla conc	NA	NA	NA
N	6	6	7
**R^2**			
Null	NA	NA	NA
Ω_{ar}	0.69	0.69	0.69
Chla conc	0.11	0.15	0.26
Ω_{ar} + Chla conc	NA	NA	NA

Supplementary Table 13: Model output of phytoplankton concentrations (chla) from the Washington and Oregon area (the model coverage) at different depths (surface, 30 m, 100 m) with associated standard deviation. The average chla concentrations from 30 and 100 m depth are derived using tracking particles model run from 1 July to 31 August 2013, while the surface chla is *in situ* chla derived for the same period of time and area.

station	lat	lon	ave chla (100m)	chla (100 m) std	ave chla (30m)	chla (30 m) std	ave chla (surface)
26	48.14	234.8	10.41	0.18	14.10	0.27	10.03
28	47.13	233.9	11.38	0.16	15.47	0.26	2.56
35	47.34	235.3	9.52	0.33	12.38	0.73	4.47
37	46.12	235.3	10.39	0.25	13.93	0.41	4.29
38	46.12	235.1	11.56	0.07	15.95	0.17	3.23
47	44.65	235.4	9.02	0.41	11.25	0.84	16.09
50	44.66	234.9	11.32	0.17	15.73	0.42	4.09
99	44.65	-124.77	NA	NA	NA	NA	11.95
104	44.65	235.9	8.76	0.03	10.06	0.06	28.74

Carbonate chemistry data: Collection, experimental conditions, and analyses

For the 2013 West Coast Ocean Acidification cruise (WCOA2013; 1–28 August 2013) CTD and oxygen sensor profile data were collected along 10 cross-shelf transects accompanied by biological stations (Fig. 1) with accompanied vertical sections of oxygen, calculated pCO_2, pH, and calculated Ω_{ar} (Supplementary Fig. S1, Table S1). Most environmental parameters were highly correlated among the stations (Supplementary Fig. S1 and Table S1). Principal component (PC) analysis was used to correlate environmental parameters at each station (Table S2).

At each station, water samples were collected in modified Niskin-type bottles, poisoned with $HgCl_2$ and analyzed after the cruise for dissolved inorganic carbon (DIC), total alkalinity (TA), and nutrients. The DIC concentration was determined by gas extraction and coulometry using a modified Single-Operator Multi-Metabolic Analyzer, with a precision of ± 1.5 µmol kg^{-1}. Seawater TA was measured by acidimetric titration, employing the open-cell method described by Dickson et al.[1], with a precision of ± 2.0 µmol kg^{-1}. Replicate samples were typically taken for two sample depths at each station and were interspersed throughout the station for quality assurance. No systematic differences between the replicates were observed. Data accuracy was confirmed by regular analyses of Certified Reference Materials[1].

In the high CO_2 experimental conditions, onboard ship seawater was collected at station 26 for the first set of experiments (Treatment 1) and at station 105 for the second set of experiments (Treatment 2). Ambient seawater was pre-conditioned with CO_2 gas to reach targeted levels of 400, 800, and 1200 µatm (within ± 5%) at 10°C when the experiments started.. CO_2 gas was continuously added to the treatments and checked for consistency with pH measurements (Supplementary Table S7) using Durafet probes. An additional (pre-conditioned)

carboy that did not contained any pteropods was bubbled with the same high CO_2 conditions and kept at the same temperature with pre-checked conditions, provided water that was circulated around other six carboys was kept was a control of carbonate chemistry conditions. On the each level of pCO_2 treatment (400, 800 and 1200 µatm), there were 6 bottles with recirculating water between them to eliminate any bottle effect. 400 µatm pCO_2 level acted as a control treatment to which 800 µatm and 1200 µatm were compared.

Using the CO2SYS software by Lewis and Wallace[2], carbonate ion concentration was calculated using carbonic acid dissociation constants of Lueker et al.[3]. The in situ degree of saturation of seawater with respect to aragonite, Ω_{ar}, is the ion product of the concentrations of calcium and carbonate ions, at the in situ temperature, salinity, and pressure, divided by the apparent stoichiometric solubility product (K'_{spar}) for those conditions, where Ca^{2+} concentrations are estimated from the salinity, and carbonate ion concentrations are calculated from the DIC and TA data (Supplementary Equation (1)):

$$\Omega_{ar} = \left[Ca^{2+}\right][CO_3^{2-}] \;/\; K^{*}_{sp_{arg}} \quad (1)$$

The temperature and salinity effect on the solubility is estimated from the equation of Mucci[4] and includes the adjustments to the constants recommended by Millero[5]. Carbonate chemistry measurements of the upper 100 m water column were not spaced evenly through the water column at each sampling station; therefore, the data were interpolated on a regular grid before the calculations of weighted averages were determined.

Pteropod collection

Pteropods were collected at the stations along the coastal, near- and offshore environment from Washington to Northern and Central California portions of the WCOA2013 cruise on *Fairweather* and *Point Sur*. The stations were characterized based on the difference in the carbonate chemistry conditions (Fig. 1 and Supplementary Fig. S1). We used a 200 µm Bongo net obliquely towed from 20 to 30 minutes in the upper 100 m. The towing duration and depth were based on previous experiences and were set to provide enough pteropods for all intended analyses. Although we collected several pteropod species, we focus only on *Limacina helicina* in this study.

Shipboard experiments*:* Determining sites of active calcification

Calcein (Bis[*N*,*N*−bis(carboxymethyl)aminomethyl]fluorescein) (Sigma, USA) is a fluorescent dye in a powdered form. Preparing calcein solution followed the protocol by Moran (2000). Powdered calcein was dissolved in water to concentrated stock solution (6.25 g/L) which was buffered with sodium bicarbonate to increase pH. This solution that was subsequently diluted to 150 mg/L (see below) for the experimental purposes, minimizing the effect of calcein on the overall pH solution in which pteropods were kept. Combined with seawater, it forms a non-toxic stock solution, where immersion into calcein produces a fluorescing mark upon incorporation into the shell matrix[6]. The incorporation of calcein allows the determination of areas of shell growth[7–9]. Prior to ship experiments, we have conducted preliminary experiments with and without calcein at control and high CO_2 treatment. The study was used as a control of the calcification treatment, making sure that calcein did no negatively affect organisms or impact their survival (no survival impact was detected) and to eliminate any artefacts due to auto-fluorescence of organisms.

The purpose of this experiment was not to measure growth only at the apertural margin (*sensu* refs. 7–9), but also the thickening that occurs simultaneously over entire or partial shell surfaces (discussed by ref. 9). For the staining, we experimented with different durations and concentrations of calcein, taking into account the size and age of individuals. Overall, we established that the most efficient concentration of calcein was 150 mg/L for approximately 18-20 hours, similar to previous studies that have used calcein for tagging (e.g., ref. 10 and references therein, ref. 11). After the staining was completed, we thoroughly washed animals three times with filtered seawater to prevent passive integration of calcein into the shell.

To determine calcification activity in response to in situ carbonate chemistry conditions, we collected and examined 96 pteropod from 12 different stations (Supplementary Table S3). At each station, 10–15 captured pteropods were kept in the water of their origin (Supplementary Table S3), to which calcein was added for 20–24 hr. This procedure allowed the pteropods to acclimatize as well as overcome any stress after capturing. Measurements of active calcification were conducted in 4 L carboys from locally collected seawater (Supplementary Table S3). Pteropods from each location were placed in a single carboy. We measured TA and DIC of the water immediately after retrieval, and measured carbonate chemistry before and after the calcein was added to it, with insignificant difference to the measurement. We used the size cohort of juvenile organisms with comparable sizes (ranging from 300 to 500 μm) to exclude the size effect. In addition, all sizes were subsequently normalized for the difference in shell size.

After exposure, pteropods were frozen to -20°C and subsequently analyzed under a compound epifluorescent microscope (Nikon Eclipse 500). We determined calcification by quantifying fluorescence mean intensity, percentage glow, and the intensity of edge glow

(Supplementary Fig. S4). We recorded the size of each examined pteropod and normalized it against all the calcification parameters.

Calcification image analysis

Our preliminary analyses showed that the organic matter retained in the body can pose an artifact in determining the extent of fluorescence, not only because it creates a much stronger signal of calcein fluorescence than at the shell surface, but also because the differentiation of the signal coming from the organics or from the shell is not possible unless we remove the organic material, to account for the epifluorescence signal (Supplementary Fig. S3). The most efficient, fastest, and easiest method to remove organics was found to be incubation of pteropods in 6% sodium hypoclorite (bleach) for 3 hours. Although we experimented with proteinase enzymes and 6% peroxide, neither of these techniques was rapid enough to prevent the weakening of the shell before the sample was examined for the fluorescence. After the removal of organics was completed, all pteropods were washed with distilled H_2O, immersed in 3% methlycellulose and positioned with the whorl facing upward. Photographs were taken at 515 nm to capture the calcein uptake in the shell where the calcein undergoes fluorescence. In some cases, the organics still remained in the very last whorl, which could create a false fluorescence signal. However, by taking an additional photo of each animal at 375 nm, we ensured that the organics did not compromise the fluorescence determination (Supplementary Fig. S3).

Our objective was to determine whether the water chemistry of origin of the collection influenced the organisms' ability to calcify. Statistically, we address this question in several steps. First, we analyzed epifluorescent images of pteropods using the Image J software package

by determining the total area of calcification and differentiating organic matter within. For the former, we used the wavelength of 515 µm to excite the calcein within the shell to fluoresce and 375 µm to determine the content of organic material (Supplementary Fig. S3). The fluorescence of the organic matter increases the total mean intensity and area of calcification; thus, it needs to be subtracted from the overall calcification. Total surface area was measured, upon which the images were converted into an 8-bit color scale using a standardized threshold for all the images, with the fluorescence of the organic material subtracted from the total calcification area to determine three metrics of calcification: total calcification area, the extent, and the mean intensity of fluorescence within the shell. Total mean intensity and edge glow were calculated by subtracting the product of organic mean intensity and the percent area of organics from the product of fluorescence mean intensity and the percent area fluorescing and at the growing edge, respectively.

Percent Calcification = Total Calcification – Total Organics (2)

Total mean intensity = (Calcification Mean Intensity * Percent Area Calcified) - (Organics Mean Intensity * Percent Area Organics) (3)

Shipboard experiments: Survival probability

To determine survival in pteropods collected from different locations at a variety of high CO_2 experimental conditions, we placed captured pteropods in shipboard flow-through aquaria in to which we bubbled CO_2 to achieve target levels of ~400, 800, or 1200 µatm pCO_2 at each level. We worked with 737 individuals from 10 different stations characterized by unique carbonate chemistry (Supplementary Table S7, S8, S10). Water was constantly bubbled into a separate carboy and this pre-acclimatized water flowed to the crate with 6 carboys at each pCO_2 level

where it was being recirculated for the whole duration of the experiment. The conditions for each individual pteropod per carboy and treatment with are presented in Supplementary Table S7.

Experiments were conducted as two separate set-ups, each for a duration of 7 days, upon which the experiments were stopped and pteropods were either frozen (for calcification analyses) or preserved in 90% ethanol (for other analyses). Experiments were conducted with up to 20 juvenile pteropods in each 4 L carboy. We treated filtered seawater with 1.7 mg/L tetracycline antibiotic to prevent bacterial infection. In addition, we added nutrients in one spike over the 3-day culturing period with Shellfish Diet 1800, a commercially prepared mix of four marine microalgae (*Isochrysis* sp, *Pavlova* sp, *Thalossiosira weissflogii*, and *Tetraselmis* sp, with the concentration of 50 µl/L, which is >50,000 cells ml^{-1}. According to Howes et al.[12], mixed algal diet at high concentration (>50,000 cells ml^{-1}) is recommended for long-term pteropod survival in experimental conditions. Success of feeding was observed by determining the fullness of guts, demonstrated by the extent of brown coloration in the guts area. After the experiments were completed, pteropod survival was determined under the light microscope based on the presence of heart beat and movement. We confirmed that food treatment did not negatively impact survival and did not impact carbonate chemistry conditions.

Statistical analyses

Analysis of environmental variables

Environmental variables were all highly correlated among stations (Supplementary Fig. S2 and Table S1, Tabe S2). We used principal components (PC) and cluster analyses[13] to more fully examine relationships among stations in water column chemistry. Water chemistry variables

were temperature (°C), salinity, dissolved O_2, DIC, TA, CO_3^{2-}, PO_4, nitrate, NH_4, pH, pCO_2, and aragonite saturation state (Ω_{ar}). Variables were normalized prior to analysis by subtracting the mean and dividing by the standard deviation to place them on the same scale[14]. The first and second PCs both had eigenvalues greater than 1.0, indicating that they explained a significant portion of the variance. However, the remaining PCs had eigenvalues below 1.0 and are not considered further[13]. Chlorophyll concentrations from observations were only available at three different stations of the first leg of the cruise, hence modeled outputs for chlorophyll concentrations were used (Supplementary Table 13).

For cluster analysis, we used hierarchical clustering with Ward's distance (squared) and a Euclidean similarity matrix. PC and cluster analyses were completed in R 3.1.1 (ref. 15) using the 'princomp' and 'hcluster' procedures, respectively. Stations 26, 28, 35, 37, 38, 47, 50, 99, 104, 105, 106, 108, and 113 were included in the analysis. For some of the stations, either calcification or survival were analyzed (but not both); we produced a common clustergram for all stations based on which four groups were formed.

Calcification statistical analyses

Three metrics of calcification were used to localize the patterns and quantify the intensity and the extent of calcification. For that, mean intensity of the fluorescence of the shell, proportional glow, and intensity of the fluorescence of the growing edge was measured. Raw data for each individuals with three different metrics is presented in Supplementary Table S6. To examine if three metrics comparatively showed the same trends and if one metric could predict the others, we used generalized additive models (GAMs) to examine the relationships between proportional glow and (1) mean intensity, and (2) edge intensity (Supplementary Fig. S5, Table S5). Since initial scatter plots showed nonlinear relationships, we used a logit-link and beta error

distribution to model the data being in the range (0–1). Because several 0s and 1s were present in the proportional glow and edge intensity data sets, we applied the following transform:

$$y_{transformed} = (y*(n\text{-}1)+0.5)/n,$$

where n is the sample size[16]. Since all three metrics were correlated and proportional glow was a good predictor of mean intensity and edge intensity (Supplementary Fig. S5), we conducted further analyses only using proportional glow. By determining the location and the extent of calcification area, proportional glow is also the most suitable way of interpreting the overall calcification process, which supports our decision to use it as a single metrics variable in further analyses.

We then used generalized linear models (GLMs, beta error distribution and logit-link, a.k.a beta regression)[17] to establish how proportional glow differed among stations and to estimate the mean and variance at each site. We compared the model Akiake's Information Criterion (AIC) versus the null model (no station term) to assess the fit of the model.

Next, we used the predicted means from the proportional glow ~ station model as the response variable for a second round of GLMs (with logit-link and beta distribution) to determine which environmental parameters (such as Ω_{ar}, temperature, and TA) predicted variation among stations in proportional glow (Supplementary Table S6). We chose these parameters because of their direct link to calcification in pteropods. Other variables that were highly correlated with Ω_{ar} were excluded from the analyses (see *Results*). We included $1/\sigma^2$ for each station mean as weights in the analysis where $\sigma^{2} = \mu\ (1\text{-}\mu)/(1+\phi)$ to account for uncertainty in the estimate of the mean. To control for multicollinearity, we first regressed (separately) TA, temperature, and pH against Ω_{ar}. We then used the residuals of these relationships as predictors in the main analysis. We fit a series of nested models (Supplementary Table S4) and selected the

best-fit model using AIC weights[18] (w_i). To avoid using the residuals, we omitted the parameters with the residuals and selected the model only using Ω_{ar} (see equation in *Results*). Beta regressions were run in R 3.1.1 using the 'betareg' package[15].

Survival statistical analyses

Our aim was to determine whether the water chemistry in the original environment influenced probability of survival at a range of experimental pCO_2 treatments in the lab. Statistically, we address this question in several steps.

First, we used a GLM with logit-link and binomial error distribution with experimental lab pCO_2 and station identity as a fixed categorical factor to determine whether survival varied among stations and pCO_2 treatments (Supplementary Tables S8, S9 and S10). Data were success (1) or failure (0) of survival for each individual pteropod. There were approximately 15–20 pteropods per station at each treatment (Supplementary Table S7).

Second, we fit a series of nested models, including the full model with an interaction between lab pCO_2 and station, no interaction, each factor individually, and a null model (intercept only). We then compared AICc values to select the best model (Supplementary Table S9, S10).

Finally, we regressed the predicted means from the best model against in situ aragonite concentration and temperature, total alkalinity, and pH residuals. Since there was no interaction term, we use the means for the 400 pCO_2 treatment for comparison because in the absence of an interaction between stations and pCO_2 treatment, the effect of station (and associated environmental variables) will be consistent across pCO_2 levels. We included 1/s.e. of the

predicted mean as a weight in the analysis. As previously, we fit a series of nested models and selected the best model based on AICc values (Supplementary Table S11).

Float tracks to determine pteropod movement

To determine the probable prior history of exposure to undersaturated conditions by the pteropod samples, we used a high-resolution hindcast model simulation of ocean conditions[19] to track particles released along transects between 44.5 and 48°N coincident with sampling locations. The particles experienced diel vertical migration between 10 and 100 m to mimic the pteropod behavior[20]. Particles were released at survey locations on 1 August 2013, and tracked one month backward (to 1 July 2013) and one month forward (to 31 August 2013) in time. Any particles entering coastal regions shallower than 100 m were constrained to remain at least 5% of the total water column thickness below the surface and above the bottom. Correlation between in situ conditions and model output (‘undersaturation days’) is shown in Fig. S6.

After particle tracks were computed, we utilized daily average fields of aragonite undersaturation calculated from a regional model of the US Pacific Northwest[19] to understand the range of saturation conditions the particle tracks experienced in their recent history. The Regional Ocean Modeling System (ROMS; Rutgers version 3) is configured for the Washington and Oregon coasts after Giddings et al.[21] using the Cascadia domain. This domain, seen in Fig. 3, extends from 43 to 50°N including Puget Sound, with a horizontal resolution of 1.5 km and 40 vertical levels. This implementation of ROMS includes 17 rivers forced with daily river temperature data from USGS gauging stations, as described by Giddings et al.[21]. The rivers enter the domain with constant saturated values of oxygen and a seasonal cycle for nutrients from a climatology of USGS gauging station data with tides included, described by Davis et al.[22]. The biogeochemical (NPZD and oxygen) model has been validated regionally[22–24]. An empirical

relationship using temperature and oxygen[25] to calculate aragonite saturation was applied to the regional simulations of oxygen and temperature to produce the aragonite saturation fields. The modeled spatial variability of the percentage of the upper 100 m that is undersaturated compares well with observations[19] (R^2=0.73).

Saturation history values that were related to shell dissolution (Supplemantary Figure 7) were derived as follows. At each field sampling station we released a 10km x 10km grid of individuals (100 x 100 array, with 0.1 km spacing) on the date of sampling, and tracked all these individuals backward in time for 30 days from the sampling date. We imposed diurnal vertical migration between 100m and 0m depth, and required that individuals stay 5m above the bottom at all times.

Supplementary Information References

1. Dickson, A. G., Afgan, J. D. & Anderson, G. C. Reference materials for oceanic CO_2 analysis: A method for the certification of total alkalinity. *Mar. Chem.* **80**, 185–197 (2003).

2. Lewis, E. & Wallace, D. W. R. Program developed for CO_2 system calculations. Tech. Rep. ORNL/CDIAC-105 (Carbon Dioxide Information Analysis Center, Oak Ridge National Laboratory, US Department of Energy, 1998).

3. Lueker, T. J., Dickson, A. G. & Keeling, C. D. Ocean pCO_2 calculated from dissolved inorganic carbon. *Mar. Chem.* **70**, 105–119 (2000).

4. Mucci, A. The solubility of calcite and aragonite in seawater at various salinities, temperatures, and one atmosphere total pressure. *Am. J. Sci.* **283**, 781–799 (1983).

5. Millero, F. J. Thermodynamics of the carbon dioxide system in the oceans. *Geochim. Cosmochim. Acta* **59**, 661–677 (1995).

6. Moran, A. L. Calcein as a marker in experimental studies newly-hatched gastropods. *Mar. Biol.* **137**(5–6), 893–898 (2000).

7. Comeau, S., Gorsky, G., Jeffree, R., Teyssié, J. L. & Gattuso, J.-P. Impact of ocean acidification on a key Arctic pelagic mollusc (*Limacina helicina*). *Biogeosciences* **6**(9), 1877–1882 (2009).

8. Comeau, S., Gorsky, G., Alliouane, S. & Gattuso, J.-P. Larvae of the pteropod *Cavolinia inflexa* exposed to aragonite undersaturation are viable but shell-less. *Mar. Biol.* **157**(10), 2341–2345 (2010).

9. Lischka, S., Büdenbender, J., Boxhammer, T. & Riebesell, U. Impact of ocean acidification and elevated temperatures on early juveniles of the polar shelled pteropod *Limacina helicina*: Mortality, shell degradation, and shell growth. *Biogeosciences* **8**, 919–932 (2011).

10. Thabet, A. A., Maas, A. E., Lawson, G. L. & Tarrant, A. M. Life cycle and early development of the thecosomatous pteropod *Limacina retroversa* in the Gulf of Maine, including the effect of elevated CO_2 levels. *Mar. Biol.* **162**, 2235–2249 (2015).

11. Reusch, T. B. H. Differing effects of eelgrass Zostera marina on recruitment and growth of associated blue mussels Mytilus edulis. *Mar. Ecol. Prog. Ser.* **167**, 149–153 (1998).

12. Howes, E. L. *et al.* Sink and swim: a status review of thecosome pteropod culture techniques. *J. Plankton Res.* **36**, 299–315 (2014).

13. Tabachnick, B. G. & Fidell, L. S. *Using Multivariate Statistics*, ed. 3 (Harper Collins College Publishers, 1996).

14. Clarke, K. R. & Warwick, R. M. *Change in Marine Communities: An Approach to Statistical Analysis and Interpretation*, ed. 2 (Natural Environment Research Council, Plymouth Marine Laboratory, Plymouth, UK, 2001).

15. R Core Team *R: A Language and Environment for Statistical Computing* (R Foundation for Statistical Computing, 2014).

16. Smithson, M. & Verkuilen, J. A better lemon squeezer? Maximum-likelihood regression with beta-distributed dependent variables. *Psychol. Methods* **11**, 54–71 (2006).

17. McCullagh, P. Regression models for ordinal data. *J. Roy. Stat. Soc. Ser. B* **42**, 109–142 (1980).

18. Burnham, K. P. & Anderson, D. R. *Model Selection and Multimodel Inference: A Practical Information-Theoretic Approach* (Springer-Verlag, 1998).

19. Siedlecki, S. A. *et al.* Experiments with seasonal forecasts of ocean conditions for the northern region of the California Current upwelling system. *Sci. Rep.* **6**, 27203; 10.1038/srep27203 (2016).

20. Bednaršek, N. & Ohman, M. D. Changes in pteropod distributions and shell dissolution across a frontal system in the California Current System. *Mar. Ecol. Prog. Ser.* **526**, 93–103 (2015).

21. Giddings, S. *et al.* Hindcasts of potential harmful algal bloom transport pathways on the Pacific Northwest coast. *J. Geophys. Res.* **119**, 2439–2461 (2014).

22. Davis, K. A. *et al.* Estuary-enhanced upwelling of marine nutrients fuels coastal productivity in the U.S. Pacific Northwest. *J. Geophys. Res.* **119**, 8778–8799, doi:10.1002/2014JC010248 (2014).

23. Banas, N. S. *et al.* Planktonic growth and grazing in the Columbia River plume region: A biophysical model study. *J. Geophys. Res.* **114**, C00B06 (2009).

24. Siedlecki, S. A. *et al.* Seasonal and interannual oxygen variability on the Washington and Oregon continental shelves. *J. Geophys. Res. Oceans* 120, 608–633, doi:10.1002/2014JC010254 (2015).

25. Alin, S. R. *et al.* Robust empirical relationships for estimating the carbonate system in the southern California Current System and application to CalCOFI hydrographic cruise data (2005–2011). *J. Geophys. Res.* **117**, C05033 (2012).
